# Biological and Genomic Characterization of a Novel Jumbo Bacteriophage, vB_VhaM_pir03 with Broad Host Lytic Activity against *Vibrio harveyi*

**DOI:** 10.3390/pathogens9121051

**Published:** 2020-12-15

**Authors:** Gerald N. Misol, Constantina Kokkari, Pantelis Katharios

**Affiliations:** 1 Institute of Marine Biology, Biotechnology and Aquaculture, Hellenic Center for Marine Research, 71500 Heraklion, Crete, Greece; gerald.san89@gmail.com (G.N.M.J.); dkok@hcmr.gr (C.K.); 2 Department of Biology, University of Crete, 71003 Heraklion, Crete, Greece

**Keywords:** jumbo phage, *Vibrio harveyi*, antibiotic resistance, phage therapy aquaculture

## Abstract

*Vibrio harveyi* is a Gram-negative marine bacterium that causes major disease outbreaks and economic losses in aquaculture. Phage therapy has been considered as a potential alternative to antibiotics however, candidate bacteriophages require comprehensive characterization for a safe and practical phage therapy. In this work, a lytic novel jumbo bacteriophage, vB_VhaM_pir03 belonging to the *Myoviridae* family was isolated and characterized against *V. harveyi* type strain DSM19623. It had broad host lytic activity against 31 antibiotic-resistant strains of *V. harveyi, V. alginolyticus, V. campbellii* and *V. owensii.* Adsorption time of vB_VhaM_pir03 was determined at 6 min while the latent-phase was at 40 min and burst-size at 75 pfu/mL. vB_VhaM_pir03 was able to lyse several host strains at multiplicity-of-infections (MOI) 0.1 to 10. The genome of vB_VhaM_pir03 consists of 286,284 base pairs with 334 predicted open reading frames (ORFs). No virulence, antibiotic resistance, integrase encoding genes and transducing potential were detected. Phylogenetic and phylogenomic analysis showed that vB_VhaM_pir03 is a novel bacteriophage displaying the highest similarity to another jumbo phage, vB_BONAISHI infecting *Vibrio coralliilyticus*. Experimental phage therapy trial using brine shrimp, *Artemia salina* infected with *V. harveyi* demonstrated that vB_VhaM_pir03 was able to significantly reduce mortality 24 h post infection when administered at MOI 0.1 which suggests that it can be an excellent candidate for phage therapy.

## 1. Introduction

The financial losses in aquaculture due to outbreaks of bacterial diseases are estimated to be in the range of billion US dollars globally. Disease outbreaks are among the most important threats for the economic sustainability of the aquaculture sector [1,2]. An important bacterial pathogen in aquaculture is *Vibrio harveyi,* which is a halophilic Gram-negative bacterium causing vibriosis disease in marine finfish, crustacean and molluscan species [3,4]. *Vibrio harveyi* is ubiquitous in the aquatic environment and can survive without a host. It is an opportunistic pathogen that will induce disease when the water temperature is optimal for its growth and at the same time its hosts are stressed [5]. *Vibrio harveyi* has also been increasingly reported in the Mediterranean aquaculture [6,7,8]. Intensification of aquaculture has been regularly considered as a major contributing factor to the outbreaks. In addition, the rising of the seawater temperature globally and climate change have also been associated with increasing *Vibrio* spp. detection in the environment [9,10,11,12]. Therefore, there is a high risk of more vibriosis outbreaks in the future. Antibiotics such as tetracyclines, fluoroquinolones and beta-lactamases have been extensively used and, in some instances, misused (or abused) in aquaculture as prophylactic or therapeutic means to control vibriosis [13]. As a consequence, resistant bacterial subpopulations develop rapidly in aquaculture through the exposure to subclinical dosages of antibiotic residues [14,15,16,17,18,19]. It is essential to reduce drastically the use of antibiotics in aquaculture or to be considered as a last resort option. To control bacterial diseases in aquaculture with reduced reliance on antibiotics, a working alternative is urgently needed. Bacteriophages or phages are ubiquitous viruses that exclusively infect bacteria. Recently, phage therapy has been revisited as a potential alternative to control bacterial diseases in aquaculture [20,21,22]. Phages are also the most abundant and diverse biological entities on earth therefore, finding phages that infect a specific strain of bacteria is relatively easy. Recent experimental phage therapy studies have also demonstrated positive results in controlling important bacterial fish pathogens such as *Flavobacterium psychrophilum* and *Vibrio* spp. [23,24,25,26,27]. However, phage therapy requires comprehensive knowledge of the applied bacteriophages therefore a full characterization of their biological and genomic attributes that includes their infectivity, lifestyle, stability, and possible virulence and antibiotic resistance encoding genes [28]. This study aimed to the isolation and characterization of a lytic phage against *V. harveyi* that could be used for phage therapy in aquaculture.

## 2. Results

### 2.1. Isolation and Morphology of vB_VhaM_pir03

**Figure 1:**
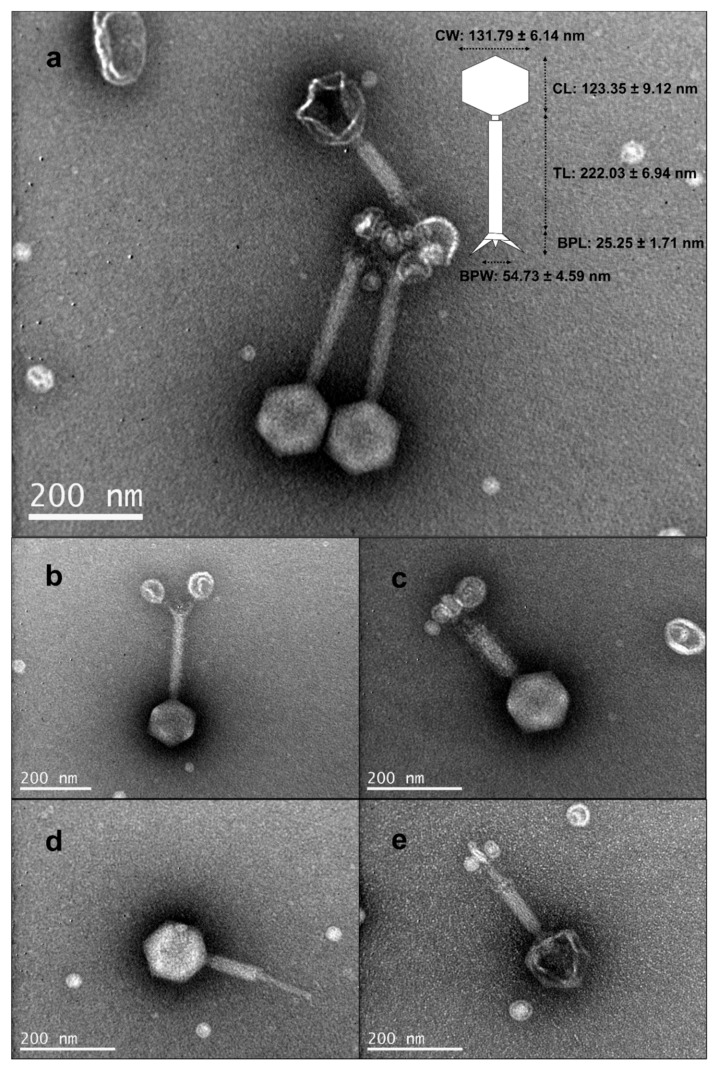
Electron micrograph of vB_VhaM_pir03: (**a**) virion morphology and dimensions—CW, capsid width; CL, capsid length; TL, tail length; BPL, baseplate length; and BPW, baseplate width; (**b**) uncontracted tail; (**c**) contracted tail sheath; (**d**) tail tube exposed; (**e**) absence of genetic material in the capsid.

vB_VhaM_pir03 was isolated from an environmental sample collected from the Port of Piraeus, Athens, Greece against *Vibrio harveyi* type strain DSM19623. A single plaque of vB_VhaM_pir03 was carefully isolated and purified through six times propagation. Throughout the propagation steps, vB_VhaM_pir03 showed a consistent plaque morphology. In the double layer agar plating assay, vB_VhaM_pir03 produced a pinhole-type plaque formation with a diameter of 0.27 ± 0.05 mm. We found that a comparison between the use of LB agar and diluted (LB/2) agar as the bottom layer for plating showed that a higher bacteriological nutrient composition reduced the visibility and plaque size of vB_VhaM_pir03 but not the actual count (data not shown). Transmission electron microscopy (TEM) showed that vB_VhaM_pir03 has a short neck, contractile tail and an icosahedral capsid (Figure 1) which indicated that its morphology is close to the phages of the *Myoviridae* family. Structural measurements of vB_VhaM_pir03 revealed relatively large virion dimensions.

### 2.2. Host Range and Efficiency of Plating (EOP) of vB_VhaM_pir03 against Multiple Antibiotic Resistant Strains

In the host range test (Table 1), vB_VhaM_pir03 was able to infect 31 out of 51 strains used. vB_VhaM_pir03 infected 21 of the 23 strains of *V. harveyi,* three of the seven strains of *V. alginolyticus*, the single strain of *V. campbellii,* both strains of *V. owensii* and four of the ten other unidentified presumptive *Vibrio* spp. There were no susceptible strains from *V. parahaemolyticus, V. anguillarum* and *V. splendidus.* EOP of vB_VhaM_pir03 was high for eight strains of *V. harveyi,* two strains of *V. alginolyticus* and two strains of unidentified *Vibrio* spp. and moderate for nine strains. For the antibiotic susceptibility tests (Table 1), all the phage susceptible strains were determined to be completely resistant against ampicillin. Eight strains were resistant against oxytetracycline, nine strains were resistant against oxalinic acid, nine strains were resistant against florfenicol, fifteen were resistant against sulfamethoxazole/trimethoprim and five strains were resistant against flumequine. Overall, vB_VhaM_pir03 was shown to be very effective against five multiple antibiotic-resistant strains and moderately effective against six multiple antibiotic-resistant strains.

**Figure 2:**
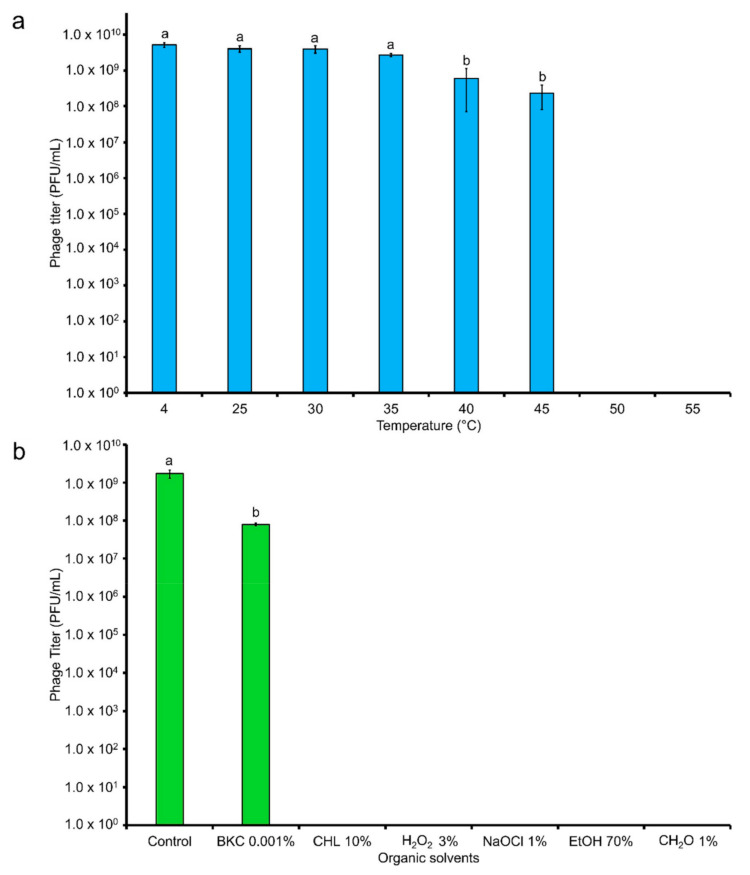
(**a**) Effect of different temperatures on the stability of vB_VhaM_pir03. Incubation at 4 °C was used as control. (**b**) Effect of organic solvents to the stability of vB_VhaM_pir03. Incubation with LB was used as control. SE bars were shown for the mean of n = 3. Statistical significance indicated by different superscript letters was determined at *p* < 0.05.

Exposure to different temperatures (Figure 2a) showed that vB_VhaM_pir03 was stable up to 35 °C. Significant reduction (one-way ANOVA, *p* < 0.05) of its titer was observed between 40 to 45 °C while complete inactivation of vB_VhaM_pir03 was observed from 50 °C and above. When exposed to 0.001% benzalkonium chloride, BKC (Figure 2b), vB_VhaM_pir03 titer was significantly reduced (one-way ANOVA, *p* < 0.05) compared to the control. However, vB_VhaM_pir03 was complete inactivated when exposed to other organic solvents.

### 2.3. Adsorption Time and One-Step Growth of vB_VhaM_pir03

**Figure 3:**
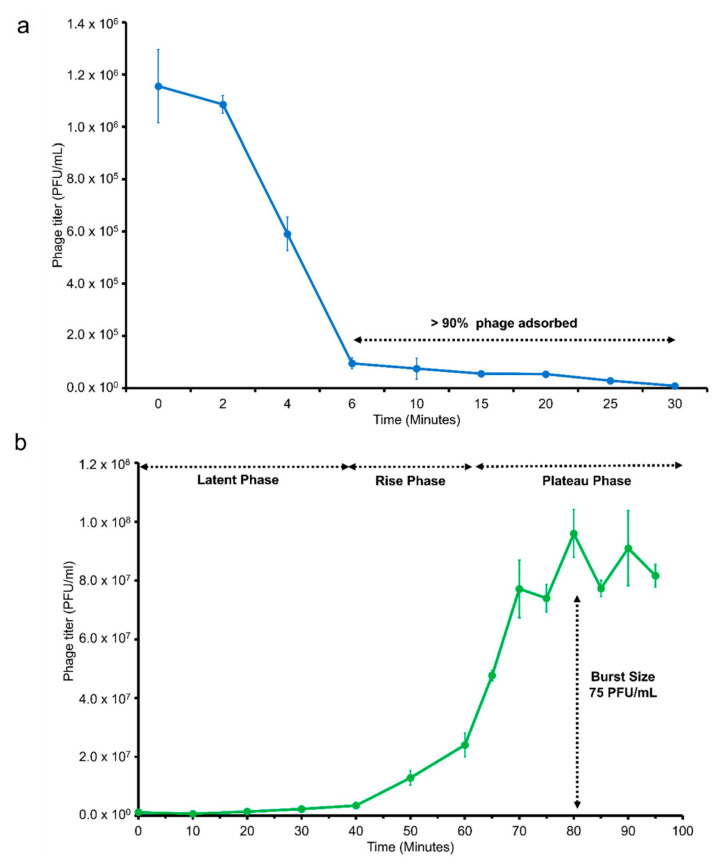
(**a**) Adsorption rate of vB_VhaM_pir03 measured against *V. harveyi* type strain DSM19623 at multiplicity of infection (MOI) 0.01. (**b**) One-step growth of vB_VhaM_pir03 measured against *V. harveyi* type strain DSM19623 at multiplicity of infection (MOI) 0.01. SE bars were shown for the mean of n = 3.

In the adsorption time assay (Figure 3a), it was estimated that the time required for 90% of the vB_VhaM_pir03 to irreversibly bind to bacterial host was 6 min. One step growth assay (Figure 3b) revealed that vB_VhaM_pir03 has a latent phase (period of between irreversible binding of vB_VhaM_pir03 to host cell until phage bursts) of 40 min. The rise phase (start of phage release from infected host until no more phages were released from its infected host) was estimated between 40 to 70 min. The plateau phase (period that indicated no more phages were released from the infected host cells) was reached at 70 min. In this assay, the burst size (number of new infective particles produced per each infected bacterial cell) of vB_VhaM_pir03 was 75 virions.

### 2.4. In Vitro Cell Lysis

**Figure 4:**
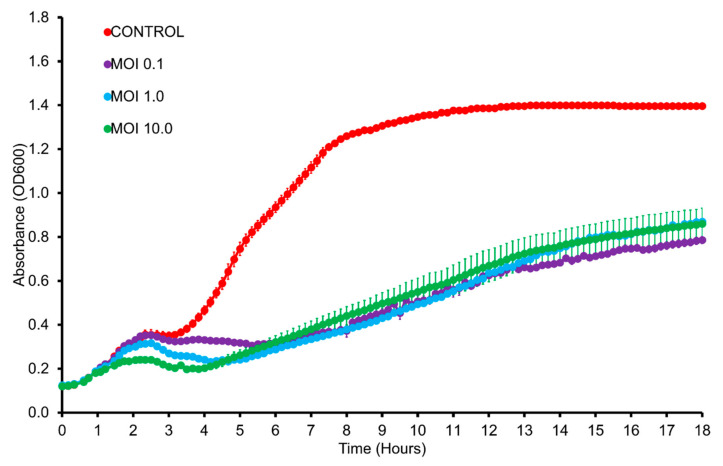
In vitro lysis of vB_VhaM_pir03 against V. harveyi type strain DSM19623 at multiplicity of infections (MOI) 0.1, 1 and 10 for 18 h. Bacterial growth indicated by the absorbance (OD_600_) read. SE bars were shown for the mean of n = 3.

In vitro lysis assay with DSM19623 (Figure 4) showed that vB_VhaM_pir03 was able to lyse the host bacterial population from MOI 0.1 to 10 after 18 h of incubation. Bacterial population infected at MOI 10 initially showed the lowest growth after 4 h of incubation but after 6 h the infected bacterial populations showed similar growth curves until 18 h of incubation to the other two MOIs used. Overall, vB_VhaM_pir03 still managed to decrease the bacterial population of DSM19623 by an approximate 40% at all MOIs compared to the uninfected population after 18 h of incubation. For in vitro lysis of vB_VhaM_pir03 with other strains, infected bacterial populations of Vh5, SerKid SA1.1, SA1.2 and SA4.1 (Supplementary Figure S1) showed similar growth patterns with DSM19623. The bacterial populations infected at MOI 10 initially showed the lowest growth however, all the infected bacterial populations eventually showed a similar growth curve pattern. For strains SNGR, VhP1 Liv, SA5.1, SA3.1, infections with vB_VhaM_pir03 with a MOI of at least 1 showed a discernible control of the host bacterial population growth. For strains Vh2, VhSerNFr, SA6.1, Vh No. 22, RG1, SA2.1, SA9.2, SA9.1, SA7.1, Art. 2, V1 and V2, infection of vB_VhaM_pir03 with MOI ≥1 was required to produce a similar outcome. However, vB_VhaM_pir03 was not effective in controlling bacterial population for strains VIB391, KS6, Barb A4/1.1, HCMR 1 Art.3, Rot. Vib. 5, Rot. 2 and Barb A4/1.2 even at MOI 10 despite those strains were susceptible in the plaque assay. The non-susceptibility of strains of Vh6 and VhKarx was also confirmed here. Finally, the bacterial population growth curves between infected and uninfected bacterial population for strains SA4.1 and Art. 2 converged when approaching 18 h of incubation.

### 2.5. Whole Genome Sequencing and Assembly

The sequenced genome of vB_VhaM_pir03 produced 41,500,540 clean reads with an average read length of 150 bp and 96.13% correct base calls. The GC content (%) was 43.6%. The per base calls scores produced good per sequence quality scores with a median of 36 for 150 bp reads. The per base sequence content and per sequence GC content showed that there was no bias in the proportion of each base position calls for four normal DNA bases or contamination during library preparation for vB_VhaM_pir03 sequencing. Finally, the per base N content result showed that no N substitutions were made which indicated that the sequencer had sufficient confidence to make base call. The genome of vB_VhaM_pir03 was assembled into a single contig with a minimum genome coverage of 5×. The total genome length of vB_VhaM_pir03 was 286,284 bp. A total of 99.91% of the raw reads were mapped back to the assembled genome resulting to an average coverage depth of 21,669×. In addition, the vB_VhaM_pir03 genome does not have any termini and was found to be terminally redundant and circularly permuted.

### 2.6. Genomic Features of vB_VhaM_pir03

The genome size of vB_VhaM_pir03 (286,284 bp) indicated that it is a jumbo phage (phages with total genome length more than 200,000 bp). The gene-coding potential of the global genome is 96.85% with 1.17 genes per kbp which suggests a dense genome arrangement. A total of 336 ORFs were identified with Rapid Annotation using Subsystem Technology (RASTk) server, 282 ORFs by Glimmer.hmm 2.0 and 286 ORFs by GeneMark. Comparison of the predicted ORFs showed that all ORFs called by Glimmer.hmm 2.0 and GeneMark were also called by RASTk. Manual inspection of each predicted ORF and gap between ORFs, and subsequent alignment in the NCBI nr database showed that 334 ORFS were present in vB_VhaM_pir03 genome. No tRNA was found in the genome. 303 ORFs used a start codon of ATG, 17 ORFs used GTG and 14 used TTG. A search on NCBI nr database showed that 119 ORFs (35.6%) had significant hits (expected value ≤10^−3^) with an average similarity of 55.8%. 71 ORFs (21.3%) were determined to have best hits with a jumbo *Vibrio* phage, vB_BONAISHI MH595538 which infects *Vibrio coralliilyticus* [29] while 20 ORFs (6.0%) had best hits with another four similar jumbo *Vibrio* phages; vB_VmeM-Yong MS31 MK308676.1, vB_VmeM-Yong MS32 MK308677.1, vB_VmeM-Yong XC31 MK308674.1 and vB_VmeM-Yong XC32 MK308675.1. In addition, protein structural homolog search for the predicted ORFs showed 26 hits in the Gene Ontology database, 35 hits with InterPro, 38 hits with the NCBI CDD and 61 hits with the HHPRED search tool. Overall, 137 (41.0%) ORFs were annotated based on amino acid sequence and protein structural homologies. No homologs of integrase, virulence or antibiotic-resistance encoding genes were found in vB_VhaM_pir03.

### 2.7. Genomic Arrangement and Functional Annotations of vB_VhaM_pir03

**Figure 5:**
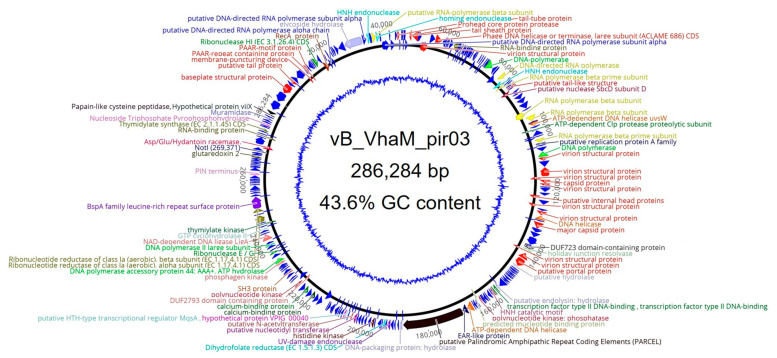
Circular representation of the vB_VhaM_pir03 genome in which the genome GC skew was shown as the inner blue circle. The predicted open reading frames (ORFs) were labelled at the outer circle. The color of the ORFs refer to annotated biochemical function; phage assembly proteins (red), DNA polymerases (green), RNA polymerases (yellow); DNA-directed RNA polymerases (blue), endolysins (grey). Other ORF colors refer to specific biochemical functions.

Generally, the genome of vB_VhaM_pir03 did not have any modular arrangement (Figure 5). However, genes encoding for head and tail proteins were arranged in subclusters while genes encoding for DNA replication and nucleotide metabolism proteins were scattered. Other genes that were functionally annotated are as in Table 2.

#### 2.7.1. Phage Structural Proteins

Proteins required for phage assembly included baseplate protein (ORF 2), tail protein (ORF 4), membrane puncturing device (ORF 8), tail-tube (ORF 51), tail-sheath (ORF 52), capsid protein (ORF 143), internal head protein (ORF 148), major capsid protein (ORF 156), portal protein (ORF 167) and other virion structural proteins (ORFs 55, 139, 141, 142, 144, 150, 152, 165 and 166). The large terminase subunit involved for DNA packaging for tailed phages was identified at ORF 57. Interestingly, Proline-Alanine-Alanine-aRginine (PAAR) repeat proteins (ORF 10 and 11), a sharp conical structure for penetration of host cells [30] were also identified adjacent to the tail, baseplate protein and membrane puncturing device proteins. In addition, a prohead core protein protease which functions to facilitate the transition of a prohead or procapsid to a mature capsid [31] was identified at ORF 42.

#### 2.7.2. DNA Replication, Repair, and Recombination

Proteins for DNA replication, recombination and repair were also identified; ribonuclease HI (ORF 16), RecA (ORF 18), HNH endonuclease (ORF 30, 107), homing endonuclease (ORF 35), DNA polymerases (ORF 101, 138, 272), SbcD nuclease (ORF 115), DNA helicases (ORF 125, 153, 198, 263), Holliday junction resolvase (ORF 164), HNH catalytic motif (ORF 185), UV-damage endonuclease (ORF 207), ribonuclease E/G (ORF 267), NAD-dependent DNA ligase LigA (ORF 275), ribonucleotide reductase (ORF 283, 286) and PIN protein (ORF 299). Glutaredoxin 2, a reducing agent for ribonucleotide reductase was identified at ORF 310. A DNA polymerase accessory protein for ATP hydrolase for DNA replication was also found at ORF 263.

#### 2.7.3. Nucleotide Metabolism and Transcription

For nucleotide metabolism, enzymes such as putative nucleotidyl transferase (ORF 223), putative N-acetyltransferase (ORF 225), thymidylate kinase (ORF 281) and thymidylate synthase (ORF 323). DNA modification enzymes were also identified such as polynucleotide kinase (ORF193) and phosphagen kinase (ORF 262). For DNA transcription, multiple RNA polymerases (ORFs 20, 29, 34, 38, 106, 109, 122, 123, 133) and RNA binding proteins (ORF 64, 319) were identified. Two ORFs encoding transcription factor type II for site-specific DNA binding [32] were identified also identified (ORF 183, 184). Although no tRNAs were found in vB_VhaM_pir03 genome, a class 2b aminoacyl-tRNA synthetases whose function is to pair tRNA with their amino acids for accurate translation of the genetic code [33] was identified at ORF 134.

#### 2.7.4. Miscellaneous Proteins

Several enzymes for lysis of host bacteria were also identified as glycoside hydrolase (ORF 28, 168, 182) which functions to degrade the host bacterial cell wall prior to phage burst. Additionally, two ORFs with no previous phage-associated descriptions were identified in vB_VhaM_pir03 genome which are ORF 200 with a structural homolog to palindromic amphipathic repeat coding elements (PARCEL) protein and ORF 64 with a structural homologs to Ro60-related proteins.

### 2.8. Genomic Synteny of vB_VhaM_pir03 with Other Similar Phages

**Figure 6:**
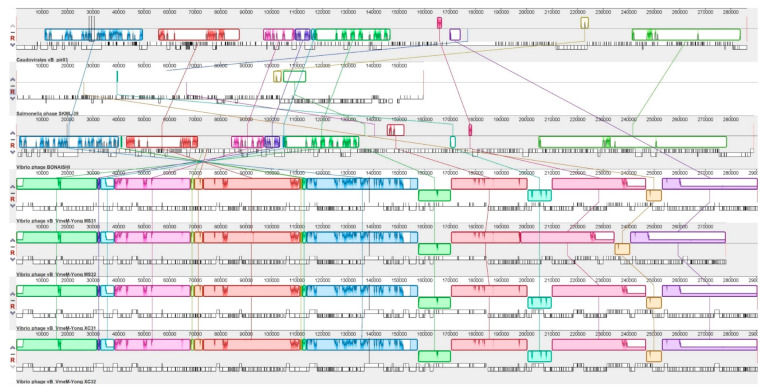
Whole genome alignment with progressive MAUVE of vB_VhaM_pir03 with similar phages. From top is vB_VhaM_pir03, *Salmonella* phage SKML-39, Vibrio phages; vB_BONAISHI, vB_VmeM-Yong_MS31, vB_VmeM-Yong_MS32, vB_VmeM-Yong_XC31 and vB_VmeM-Yong_XC32. The colored collinear blocks indicate homologous regions between genome sequences while the height of the similarity profile in the collinear blocks indicate average level of conservation in the regions of the genome sequence. Inverted blocks indicate homologous regions that align in the complement orientation.

Following whole genome alignment with the most similar phage genomes obtained from the NCBI nr database (Figure 6), vB_VhaM_pir03 was shown to have the highest degree of genomic synteny with vB_BONAISHI. Both phages also shared six collinear blocks with similar length. The longest shared collinear block had a sequence length almost 50,000 bp. Despite similar genomic arrangements, the shared collinear blocks showed very low DNA sequence similarities between them. vB_VhaM_pir03 also shared a single collinear block (<3000 bp) with *Salmonella* phage SKML 39. Alignment with another four similar jumbo phages; vB_VmeM-Yong_MS32; vB_VmeM-Yong_XC31; vB_VmeM-Yong_XC32 and vB_VmeM-Yong_MS31; showed shared collinear blocks of different length with no synteny and very low sequence similarities.

### 2.9. Phylogenetic Analysis

**Figure 7:**
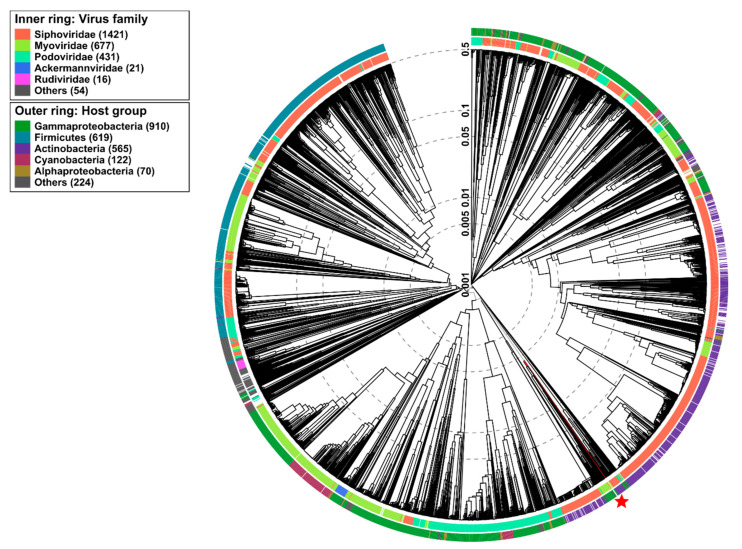
Determination of taxa and host group for vB_VhaM_pir03 by a proteomic tree using VIPTree. vB_VhaM_pir03 was determined to belong to Myoviridae family and infect Gammaproteobacteria group (red star and line). vB_VhaM_pir03 (asterisk) proteome was compared with 2688 dsDNA phages proteomes. The branch length scale was calculated as log values. The inner and outer ring indicate the taxonomic virus family and host group, respectively.

Wide genome proteomic tree analysis (Figure 7) showed that vB_VhaM_pir03 belong to the *Myoviridae* taxonomic family however, it was observed that the position of vB_VhaM_pir03 was in a subcluster within the *Siphoviridae* family. In addition, vB_VhaM_pir03 was also determined to infect a host from the *Gammaproteobacteria* class which includes *Vibrionaceae* family.

**Figure 8:**
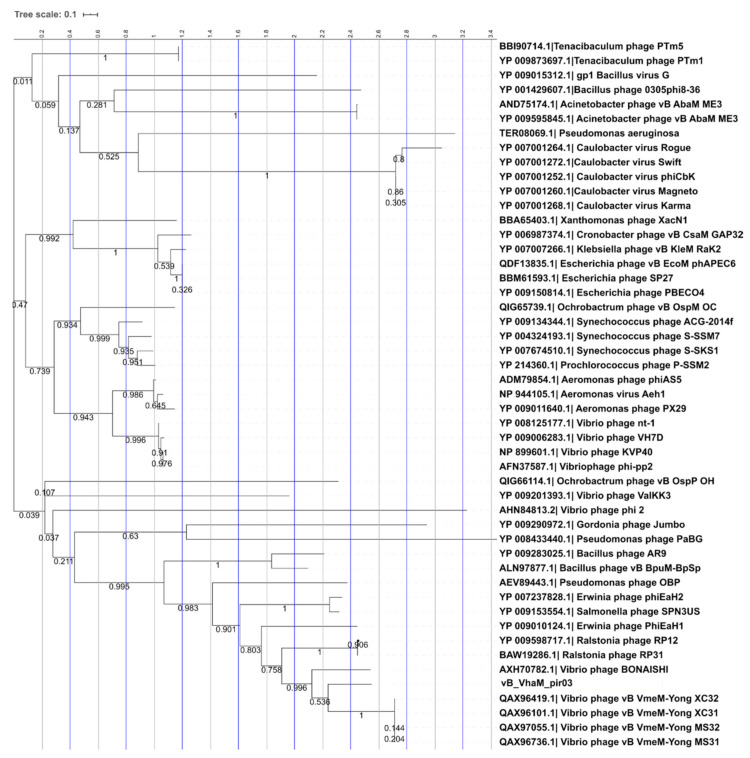
Phylogenetic tree of vB_VhaM_pir03 with other jumbo phages. The large terminase subunits of jumbo phages were downloaded from NCBI database and aligned using MUSCLE and a maximum likelihood (bootstrap = 1000) phylogenetic tree was constructed using MEGA X. The tree was visualized using Interactive Tree of Life (ITOL). The bootstrap support value was denoted in each branch.

Phylogeny using large terminase subunits of jumbo phages (Figure 8) showed that vB_VhaM_pir03 have a recent common ancestor with vB_VmeM-Yong_MS32, vB_VmeM-Yong_XC31, vB_VmeM-Yong_XC32 and vB_VmeM-Yong_MS31 although with the bootstrap value (0.536) for this inference is below the acceptable threshold of 70% for bootstrapping [34]. However, vB_VhaM_pir03 has a high bootstrap support (0.996) with vB_BONAISHI. In addition, the branch length indicated that both phages share similar number of amino acid substitutions in their large terminase subunit since diverging from their common ancestor.

### 2.10. In Vivo Phage Therapy Trial with Artemia nauplii

**Figure 9:**
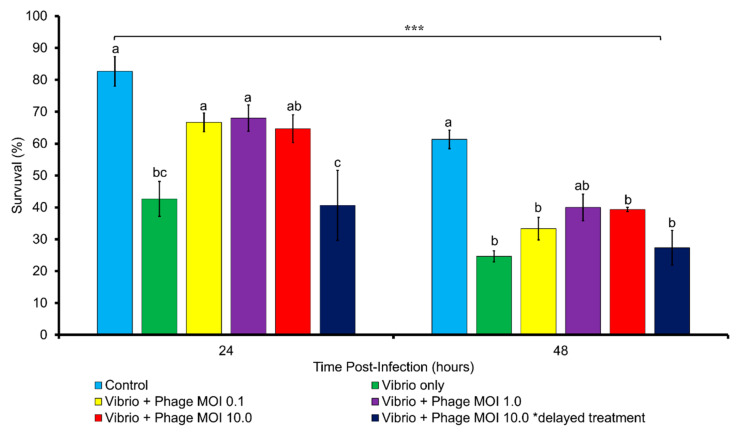
Survival of Artemia nauplii infected with Vh5 strain in an experimental phage therapy trial. Artemia nauplii infected with Vh5 were inoculated with vB_VhaM_pir03 with different multiplicities of infection (MOI) at 2 h and at 24 h of post-infection. Survival was measured at 24 and 48 h post infection. SE were shown for the mean of n = 3. Statistical differences between treatments and time post-infection were indicated by the superscript letter(s) and *** respectively (*p* < 0.05).

At 24 h post infection, the survival of the *Vibrio harveyi*-infected *Artemia* was approximately 30% which was lower than the phage-treated groups (except for MOI 10) and comparable to the untreated control group (Figure 9). At 48 h post infection, survival of the phage-treated group was higher than the *Vibrio harveyi*-infected control group although this difference was not statistically significant. The delayed treatment resulted in similar survival to the *Vibrio harveyi*-infected control group both at 24 and 48 h post infection. Measurement of the presumptive *Vibrio* load at 24 and 48 h post infection showed no significant differences (two-way ANOVA, *p* < 0.05) between all treatments (data not shown). No colony forming units were observed in the control group at both times of measurement.

## 3. Discussion

Phage therapy is a very promising alternative to antibiotics. While scientific publications about isolation of phages have increased in the last decade, there is only a small group of phages that have been applied for commercial use [35] and only one for aquaculture which is CUSTUS^®^YRS against *Yersinia ruckeri* [36]. One of the main reasons for the lack of commercialized phages is insufficient characterization which is a prerequisite for phage therapy [37]. These characterizations are necessary to identify and reduce risks associated with phage therapy which in turn, will support progress towards regulatory approval [28,38]. Therefore, these considerations were the highest priorities when performing characterization of bacteriophages in this study.

In the present study, we have isolated and characterized a novel jumbo bacteriophage, vB_VhaM_pir03 with broad host lytic activity against *Vibrio harveyi* type strain DSM19623 and analyzed its therapeutic potential for aquaculture. Transmission electron microscopy revealed that vB_VhaM_pir03 is related to the *Myoviridae* family based on the presence of an icosahedral head and long contractile tail [39]. In addition, vB_VhaM_pir03 also had relatively large structural dimensions compared to other Myoviruses infecting *Vibrio* spp. [40,41]. The main factor for large structural dimensions is still undetermined but it has been posited that a ratcheting mechanism is present for large phages especially jumbo phages for the accommodation of large genome sizes and potential acquisition of more genes [42]. In relation to plaque formation, it has also been suggested previously that large structural dimensions of phages have contributed to small plaque formations as observed for vB_VhaM_pir03 due to reduced diffusion capacity of the large virion particles [29].

Several factors affect the reproductivity and stability of phages. In our study, the significant reduction of vB_VhaM_pir03 titers was first observed at 40 °C which was lower than that of previously described *Vibrio* phages [24,25,43,44]. Poullain et al. [45] have shown that heat treatment caused damage to tailed phages such as detachment of head and tail, empty capsids, and aggregation of tails. However, since the vB_VhaM_pir03 thermal tolerance was lower than previous reports, we suggest that this phage was more sensitive due to its large genome. Previous reports [46,47] have shown that phages with large genomes have high internal capsid pressure due to dense genome packaging. These phages were demonstrated to eject their genomic material at 37 °C by the internal capsid pressures. Nevertheless, the thermal stability of vB_VhaM_pir03 do not hinder its potential direct application in aquaculture since rearing temperatures of aquatic organisms do not reach 37 °C. vB_VhaM_pir03 was also found to be completely inactivated by chloroform in the present study therefore, chloroform was not used in all assays conducted subsequently. Tailed dsDNA phages do not contain lipid membrane [48] and are not sensitive to chloroform. A recent study [49] however has reported that several tailed dsDNA phages including members of *Myoviridae* and *Podoviridae* families showed reduction in phage titer after exposure to chloroform. In addition, vB_VhaM_pir03 was also completely inactivated after exposure to organic solvents in this study except for benzalkonium chloride, BKC. This suggests that vB_VhaM_pir03 applications can be controlled for contaminations and unintentional transfers such as observed in inactivation of *Lactobacillus* phages during milk productions [50].

vB_VhaM_pir03 showed a rapid adsorption time to its host compared to previously reported *Vibrio* and jumbo phages [24,25,51,52,53]. This indicated the efficiency of vB_VhaM_pir03 to locate and irreversibly bind to the host receptors under controlled conditions. Although the main factor determining adsorption time is the rate of phage–host encounters [54], application of rigorous aeration in an aquaculture system may increase the rate of phage–host encounters by reduction of phage and host sedimentations [55]. In one-step growth assay, vB_VhaM_pir03 displayed a short latent period and high burst size similar to previously reported jumbo phages [44,56,57]. A short latent period would result in low burst sizes of phages due to limited time for viral reproduction cycles [54]. Nonetheless, vB_VhaM_pir03 was shown in this study to have an efficient viral replication mechanism which allowed high virion productions in short latent periods. As a result, a minimal concentration of vB_VhaM_pir03 can be considered in therapeutic applications in aquaculture due to its rapid adsorption time and high multiplication rate. Finally, high multiplication rate of phages has been suggested as an evolutionary trade off with low phage survivability under stressful conditions [46] which may provide an insight to the low thermal stability of vB_VhaM_pir03.

In small phages (<200,000 bp), the arrangement of core genes that encode for head, tail, DNA replication and nucleotide metabolism proteins are conserved in a modular order for maintenance of functions throughout its replication cycles [58,59] however, we found the arrangement of the core genes in the vB_VhaM_pir03 genome was generally scattered and formed subclusters as previously described by Yuan and Gao [60]. Interestingly, we also found genes in vB_VhaM_pir03 that encode multisubunit RNA polymerases (RNAPs). RNAPs are found in jumbo phages and function to trigger early DNA transcription during infection without requiring the host’s DNA machinery [61]. Furthermore, we also found genes which encode proteins that have no definitive described functions in phages. A very large gene, ORF 200 with a nucleotide length of 19,254 bp contained a protein domain termed PARCEL (Palindromic Amphipathic Repeat Coding Elements) which has not been described in phages up to date. These repeats have dyad symmetry and variable hydrophilic and conserved hydrophobic regions and have been found in bacteria, eukaryotes [62] and recently in giant viruses [63]. PARCEL protein is related to mobile elements and has been considered as products of horizontal gene transfer [64], however the GC content of ORF 200 is not different to the GC content of the phage genome, which contradicts this likelihood. It has also been suggested that PARCEL protein facilitate diversification of bacterial surface protein [62] and also play a role in the coevolution of viruses with their hosts [63]. In addition, we also found a Ro60 related protein which has been described to have Y RNAs bound to its structure. The exact functions of this protein are unknown, but several phages have been reported to have Yr1A, a protein module within Y RNA that can mimic the structure of tRNA [65]. This supports the suggestion on the diverse genetic resources contained within jumbo phage genomes as a result of the large number of gene acquisition [66].

Phages have been associated with risks of horizontal gene transfer [20] therefore, it is imperative that any phage considered for therapy are investigated for temperateness and transduction potential. In our present study, we did not find any integrase, virulence or antibiotic resistance encoding genes in vB_VhaM_pir03 genome. Hence, this phage has an exclusive lytic lifestyle and does not risk antibiotic resistance and virulence gene transfers for applications. The vB_VhaM_pir03 genome is also absent of any termini, is circularly permuted and terminally redundant which suggest a headful packaging mechanism [67]. Based on the reads mapped to the vB_VhaM_pir03 genome, the potential host sequence was ≤0.09% which indicated that vB_VhaM_pir03 does not exhibit any transduction potential [68]. From whole genome sequence homolog search, a novel jumbo phage, vB_BONAISHI which infects *Vibrio coralliilyticus*, was determined to be the only similar phage to vB_VhaM_pir03 with an average ORF similarity of 52.3%. Analysis of genomic synteny in this study revealed that both phages shared similar genomic arrangements but with low nucleotide sequence similarities. In addition, phylogenetic analysis using large terminase subunits of vB_VhaM_pir03 and other described jumbo phages produced strong bootstrap support to the evolutionary relationship between vB_VhaM_pir03 and vB_BONAISHI. This suggested that both phages may have diverged from a common ancestor but have since undergone multiple nucleotide substitution events [61]. Since, vB_BONAISHI was previously described as a singleton phage in the jumbo phage phylogenetic tree [29], the phylogenetic and phylogenomic analyses indicated that vB_VhaM_pir03 is a novel phage.

vB_VhaM_pir03 was shown to have a broad host lytic activity against different *Vibrio* species within the Harveyi clade [69,70]. To our knowledge, only one other *Vibrio* phage, KVP40 was reported to infect multiple *Vibrio* species [71]. The ability of phages to infect different strains and species of bacteria has been suggested as an adaptation tool for survival [72]. For jumbo phages, their diversity of gene functions was suggested to be responsible for their broad host lytic activity [73,74]. In our study, the broad host lytic activity of vB_VhaM_pir03 can be considered analogous to broad-spectrum antibiotics [75] therefore in aquaculture, pathogenic *Vibrio* species are ubiquitous and diverse [76,77] thus a broad host phage such as vB_VhaM_pir03 would be advantageous in controlling the *Vibrio* population. It has been previously reported that there is an inverse relationship between bacterial antibiotic and phage resistance due to the high biological cost to maintain each resistance mechanism [78]. In this study, the susceptible host strains to vB_VhaM_pir03 were determined to be multiple antibiotic resistant bacteria. Therefore, the use vB_VhaM_pir03 would be a suitable approach against antibiotic resistant bacteria either as an antibiotic alternative or as a co-therapeutant.

In vitro lysis is typically carried out as an intermediate step to large scale applications. In this step, the therapeutic effects of phages at different MOIs and the host resistance development are measured concurrently against time [38]. In the present study, several strains including the host were determined to be susceptible to vB_VhaM_pir03 suggesting that when applied *in vivo*, pharmacokinetics and pharmacodynamics of the phage therapy are the major factors in the treatment efficacy [79]. Based on the in vitro lysis assays, host bacterial growth which were inhibited by vB_VhaM_pir03 at MOIs 10 and below would be ideal for immersion type delivery method since the concentration of phage required for application in an aquaculture setting would be practical. However, for hosts needing higher concentrations of vB_VhaM_pir03, further investigations are required to determine if site-specific delivery methods such as oral or intraperitoneal injection can provide effective therapeutic effect. Nonetheless, several host bacterial strains used in the in vitro lysis showed continuous growth even after inoculation of vB_VhaM_pir03. This suggested the development of resistant bacterial subpopulation due to selective pressure [80]. This supports further investigations into co-administration of vB_VhaM_pir03 with another phage as a cocktail in which each phage utilizes different infection mechanisms to overcome bacterial phage resistance mechanisms and avoid competitive phage infection [53]. In addition, the continuous bacterial growth even after inoculation of vB_VhaM_pir03 during the in vitro lysis assays may also suggest the development of a phage carrier state population in which the phage and host exist in equilibrium without any lysogenic activity or viral replication. In a phage carrier state, the phage may reside in its host cell without any lysogenic conversion, as a possible means of protection against environmental factors and avoidance of bacterial phage resistance mechanisms [81]. While the development of phage carrier state during vB_VhaM_pir03 infections was not examined, previous studies have reported the existence of phage carrier states resulting in reduction of virulence of *Pseudomonas aeruginosa* in biofilms and new colonization of *Campylobacter jejunii* in chickens [82,83].

In the in vivo trial with *Artemia* nauplii, we found that a single dosage of vB_VhaM_pir03 was effective in increasing survival of *Artemia* nauplii infected with *Vibrio harveyi* strain Vh5 at 24 h post infection even at MOI 0.1. This result was showed that vB_VhaM_pir03 performed slightly better than a previously reported phage therapy trial with *Artemia* spp. in which the survival was measured at 50% [26] However, it was observed that vB_VhaM_pir03 was unable to provide protection to the *Artemia* nauplii at 48 h post infection. Nonetheless, *Artemia* nauplii population still showed a higher percentage of survival to the untreated population which suggested a residual effect of protection. Despite the short protective period provided by vB_VhaM_pir03, a single dosage of vB_VhaM_pir03 can also be considered as a potential live feed disinfectant since *Artemia* nauplii are fed to fish within 24 h post hatch. For the infected *Artemia* nauplii that received a delayed treatment, vB_VhaM_pir03 was unable to provide protection which suggested that the damage caused by vibriosis was irreversible similarly to the reported results of Diaz et al. [84]. Phages are conventionally used in therapeutic purposes [27], however, for pathogens which cause irreversible damage, consideration may be given to use of phages as prophylaxis such as suggested by Silva et al. and Zaczek et al. [85,86].

## 4. Conclusions and Future Directions

In this study, we have provided a comprehensive biological and genomic characterization of vB_VhaM_pir03 as a candidate for phage therapy against *Vibrio harveyi*. The biological characterization of vB_VhaM_pir03 showed that it can rapidly locate and adsorb to a host and produce a high burst size within a short latent phase. Further characterization showed that vB_VhaM_pir03 has a broad lytic activity against thirty-one multiple antibiotic resistant strains of species belonging to the Harveyi clade. This is a unique ability of vB_VhaM_pir03 which has only been reported in only one other *Vibrio* phage, KVP40. Genomic analysis revealed a wealth of diverse gene functions that contributed to the efficacy of vB_VhaM_pir03. Furthermore, we also suggest that vB_VhaM_pir03 is not a temperate phage, does not harbor virulence or antibiotic resistance genes and does not exhibit transduction potential. Evaluation of the performance vB_VhaM_pir03 in vitro showed that it can inhibit several host bacterial growths at low MOI which supports its application in phage therapy. Finally, in the in vivo trial, vB_VhaM_pir03 was able to provide some protection to *Artemia* nauplii against vibriosis at 24 h post infection at all MOIs. Further characterization of its genome to understand its underlying mechanism is suggested to be carried out. In addition, we also suggest that large scale phage therapy trial with vB_VhaM_pir03 which includes investigations into different types of delivery methods. Finally, we would also like to emphasize that phage characterizations should be comprehensive to ensure a safe and practical phage therapy. This is very important towards the progress of phage therapy from the regulatory perspective.

## 5. Materials and Methods

### 5.1. Bacterial Strains Used in This Study

Thirty-one strains of *Vibrio harveyi, V. alginolyticus, V. owensii, V. anguillarum, V. campbellii, V. parahaemolyticus* and other *Vibrio* spp. (Table 3) used in this study were obtained from the bacterial collection of the Laboratory of Aquaculture Microbiology, Institute of Marine Biology, Biotechnology and Aquaculture (IMBBC), Hellenic Center for Marine Research (HCMR) in Heraklion, Crete. The bacterial strains were previously identified either through their NCBI or ENA accession numbers for the type strains, biochemical test (BIOLOG GENiii) and PCR method (16 s rRNA and toxR amplifications). In addition, unidentified *Vibrio* spp. isolated from the live feeds of HCMR were also used. All the bacterial strains were maintained in microbeads (MicroBank) at −80 °C and were grown in Lysogeny Broth (10 g/L tryptone, 5 g/L yeast extract, 10 g/L NaCl, 1L deionized water, 0.75 g/L MgSO_4_, 1.5 g/L KCl, 0.73 g/L CaCl_2_) at 25 °C when used.

### 5.2. Antibiotic Susceptibility Testing

Antibiotic susceptibility testing was carried out for the bacterial strains used in this study according to standard disk diffusion test [87]. Bacterial suspension of the 31 selected strains were diluted to obtain a 0.7 absorbance read at OD_600_. The diluted bacterial suspensions were then plated on Mueller-Hinton agar (Difco, Detroit, MI, USA) with 2% NaCl. Antimicrobial susceptibility disks (ThermoFisher Scientific, Waltham, MA, USA) (Table 4) were placed on the agar plates and incubated at 25 °C (optimal temperature for the bacteria used) for 24 h. The recorded diameters were interpreted as susceptible, medium, or resistant according to the Clinical Laboratory Standards Institute (CLSI) guidelines CLSI M45-A2 [88] and CLSI M100-S25 [89] as in Table 4.

### 5.3. Isolation and Purification of Bacteriophages

Water samples were collected from three locations: (a) the Port of Piraeus, Athens, (b) the Karavolas beach, Heraklion, Crete and (c) a fish tank in the broodstock section of HCMR in Heraklion. 250 mL of the collected water samples were then enriched with 25 mL of concentrated LB and 2.5 mL of the host strain, *Vibrio harveyi* type strain DSM19623 liquid culture. The enriched water sample were incubated at 25 °C with a shaking speed of 70 rpm for 24 h. Following filtration through a 0.22 µm sterile filter (GVS Life Sciences, Sanford, ME, USA), 10 µL of each sample were spotted on bacterial lawns of the host strain. Following 24 h incubation at 25 ℃, the clearest plaque formations were collected. Serial propagations for phage purification were then made for the collected plaques against its host by double agar layer method according to Clokie et al. [90]. A single plaque was carefully collected, serially diluted, and propagated again in host bacterial lawn. This step was repeated at least five times for a phage to be considered purified. Phages that showed drastic decrease or loss in plaque formation during purification steps were discarded. One of the purified phages was selected for further characterization and was named vB_VhaM_pir03.

### 5.4. Transmission Electron Microscopy

For transmission electron microscopy, aliquot of vB_VhaM_pir03 suspension with a titer of ~10^10^ PFU/mL was prepared and negatively stained with 4% w/v uranyl acetate (pH 7.2). The phage was observed using a JEOL transmission electron microscopy operated at 80 kV at the Electron Microscopy Laboratory in the University of Crete. From the obtained digital micrographs, structural dimensions of individual phages were measured with ImageJ software version 1.52t [91] for capsid width, capsid length, tail length, baseplate length and baseplate width.

### 5.5. Host Range Test

For determination of host range for the purified phage, fresh cultures of bacterial strains used in this study (Table 1) were grown in LB at an approximate concentration of 10^7^ CFU/mL and were then mixed with top molten LB agar (0.75% agar) and poured on bottom LB/2 agar (5 g/L tryptone, 2.5 g/L yeast extract, 10 g/L NaCl, 1L deionized water, 0.75 g/L MgSO_4_, 1.5 g/L KCl, 0.73 g/L CaCl_2_) which only contained half of the tryptone and yeast content from the LB agar. After solidification of top agar, 10 µL of vB_VhaM_pir03 were spotted on the host lawn. The phage titer was determined after the agar plates were incubated at 25 °C for 24 h.

### 5.6. Efficiency of Plating (EOP)

Efficiency of plating (EOP) was performed in this study according to [90]. The phage was serially diluted to ~10^0^, 10^−1^, 10^−2^, 10^−3^, 10^−4^ and 10^−5^ and spotted on the bacterial lawns of the 31 susceptible strains. The phage titer was determined after the agar plates were incubated at 25 °C for 24 h. The EOP was calculated as a percentage of the number of plaque-forming units formed on a bacterial strain against the number of plaque-forming units formed on the host DSM19623. EOP more than 10 was categorized as high, EOP between 9.9 and 0.5 was considered medium while EOP less than 0.5 was considered low.

### 5.7. Stability of Phage in Different Temperatures and Organic Solvents

Phage thermal stability was measured by exposing the phage aliquots at ~10^7^ PFU/mL to different temperatures (25, 30, 35, 40, 45, 50 and 55 °C) for 1 h before being rested at 25 °C for 10 min. The aliquots were then serially diluted and spotted on host bacterial lawn. The phage titer was determined after the agar plates were incubated at 25 °C for 24 h. vB_VhaM_pir03 stored at 4 °C for 24 h was used as a control.

The sensitivity of vB_VhaM_pir03 to chloroform was determined by exposing ~10^7^ PFU/mL of the phage aliquots to 10% chloroform at 4 °C for 1 h while the stability of the vB_VhaM_pir03 against commonly used disinfectants in aquaculture was measured by exposing ~10^7^ PFU/mL of vB_VhaM_pir03 to 0.001% benzalkonium chloride, BKC; 3% hydrogen peroxide, H_2_O_2_; 1% sodium hypochlorite, NaOCl; 70% ethanol, EtOH and; 1% formaldehyde, CH_2_O at 25 °C for 1 h. vB_VhaM_pir03 incubated at 25 °C for 1 h were used as control. Each treatment was serially diluted and spotted on host bacterial lawn. The phage titer was determined after the agar plates were incubated at 25 °C for 24 h. All assays were done with triplicates.

### 5.8. Adsorption Time and One-Step Growth

Adsorption time and one-step growth of vB_VhaM_pir03 was determined according to Kutter [92] with some modifications. Briefly, host culture in exponential phase (~10^8^ CFU/mL) was infected with vB_VhaM_pir03 at multiplicity of infection (MOI) 0.01. Aliquots of the infected culture were then collected and transferred to chilled Eppendorf tubes at 0, 2, 4, 6, 10, 15, 20, 30 min, and kept in ice. The aliquots were centrifuged at 13,000 rpm for 3 min and supernatants were collected and serially diluted. The serial dilutions were then spotted on the host bacterial lawn on LB/2 agar plates. The phage titer was determined after the agar plates were incubated at 25 °C for 24 h.

For one-step growth, 1 mL of host culture in exponential phase (~10^8^ CFU/mL) was centrifuged at 13,000 rpm for 3 min. The supernatant was then discarded and the pellet was washed and resuspended in 1 mL of SM buffer (5.8 g/L NaCl, 2 g/L MgSO4, 50 mL 1M Tris-Cl pH 7.5 and 2% gelatin, 1 L deionized H_2_O). This step was then repeated twice before the pellet was finally resuspended in 1 mL of LB. The fresh host culture was then infected with vB_VhaM_pir03 at MOI 0.01. After incubation for 10 min at 25 °C, the infected DSM19623 culture was then transferred to LB with the final volume of 30 mL. Afterwards, 1 mL aliquots were then collected from the infected host culture and immediately transferred to chilled Eppendorf tubes. The aliquots were then centrifuged for 13,000 rpm for 3 min. Subsequently, the supernatants were collected and serially diluted. The serial dilutions were then spotted on the host bacterial lawn on LB/2 agar plates. This step was repeated at 10 min intervals. The phage titer was determined after the agar plates were incubated at 25 °C for 24 h.

### 5.9. In Vitro Cell Lysis

The in vitro cell lysis of vB_VhaM_pir03 against DSM19623 was carried out by loading 180 µL of fresh host bacterial culture in each well of sterile 96-well plates. The plates were then read at OD_600_ using TECAN microplate reader (Infinite PRO 200) at 25 °C with orbital shaking. A total of 20 µL of vB_VhaM_pir03 was then added at MOIs 0.1, 1 and 10 when host culture was at exponential phase (~10^8^ CFU/mL). Phages added to LB without host bacteria served as control. The assay was also carried out for the remaining 30 susceptible hosts of vB_VhaM_pir03. The growth curves of the cultures were then measured every 10 min for 18 h. All assays were done in triplicates.

### 5.10. DNA Extraction and Purification

The DNA extraction of vB_VhaM_pir03 was carried out using the phenol-chloroform method according to Higuera et al. [51]. The extracted DNA was visualized for quality on 1% agarose gel electrophoresis at 80 kV for 1 h with a 50 kbp ladder. Milli-Q^®^ Reference Water (Merck KGaA, Darmstadt, Germany) was used as a negative control. The extracted DNA of vB_VhaM_pir03was then stored in −20 °C.

### 5.11. Genomic Analysis

The whole genome of vB_VhaM_pir03 was sequenced, assembled, and annotated previously as described in Misol et al. [93]. The genome sequence of phage vB_VhaM_pir03 is available in GenBank under accession number MT811961. The associated BioProject, SRA, and BioSample accession numbers are PRJNA665717, SRR12712979, and SAMN16261552, respectively. QUAST v4.6.3 [94] and BBMap v38.88 [95] were used to map the reads back to the assembled genome while PhageTerm was used to predict phage termini [68] through the Galaxy server [96]. Proteins of vB_VhaM_pir03 were automatically annotated in Blast2GO Suite [97] using (i) NCBI Basic Local Alignment Search Tool (BLAST) [98] adjusted at non-redundant (nr) protein database, (ii) Gene Ontology [99] and (iii) InterPro [100]. Predicted proteins of vB_VhaM_pir03 were also manually annotated with NCBI Conserved Domain Database (NCBI CDD) [101] and HHpred tool [102]. All ORF predictions and annotations were manually inspected. Integrase, virulence and antibiotic resistance-encoding genes in vB_VhaM_pir03 were searched using INTEGRALL Database webserver [103], Virulence Factor Database (VFDB) [104], VirulenceFinder and ResFinder webservers [105]. The host DSM19623 genome was analyzed for prophage-like sequences using Prophinder [106] and PHAge Search Tool Enhanced Release (PHASTER) [107]. For protein structural homologies, only probabilities above 90% were accepted for manual protein function assignment of the vB_VhaM_pir03 predicted ORFs. All hits in existing databases with expected value above 10^−3^. The genome of vB_VhaM_pir03 with annotated predicted ORFs was then visualized in a circular representation with Geneious software (Geneious v9.1, Biomatters, Auckland, http://www.geneious.com).

### 5.12. Genome Alignment and Phylogenetic Analysis of vB_VhaM_pir03

The whole proteome of vB_VhaM_pir03 was searched for similarity to other phages using the NCBI BLASTP nr protein database. The phage genomes with significant similarities were then downloaded and aligned with vB_VhaM_pir03 using the progressiveMauve: Multiple Genome Alignment [108] for analysis of the genomic synteny. The viral taxonomic family of vB_VhaM_pir03 and its host taxonomic group were determined using ViPTree: the viral proteomic tree server [99]. A six-frame translation proteome of vB_VhaM_pir03 was generated and compared to other six-frame translations of dSDNA phages in the NCBI database [109]. Phylogeny and molecular evolutionary analyses with other jumbo phages were conducted with Molecular Evolutionary Genetics Analysis (MEGA X) webserver [110]. Forty-nine large terminase subunits of described jumbo phages were downloaded from NCBI database and were aligned with the large terminase subunit of vB_VhaM_pir03 using MUSCLE algorithm [111]. Gaps in the amino acid sequence alignments were trimmed. A maximum likelihood phylogenetic tree was constructed using JTT matrix-based model [112] with bootstrap test = 1000. The tree was visualized using Interactive Tree of Life web server [113].

### 5.13. In Vivo Phage Therapy Trial Using Artemia nauplii

Six different treatments were investigated to assess the efficacy of vB_VhaM_pir03 in controlling pathogenic *Vibrio harveyi* strain VH5 in brine shrimp, *Artemia salina* nauplii. The first two treatments were control groups: a negative control containing *Artemia* nauplii only and a positive control of *Artemia* nauplii with *V. harveyi*. The other three treatments were single doses of vB_VhaM_pir03 at MOI 0.1, 1 and 10. The final treatment was a group that received a delayed single dose of vB_VhaM_pir03 at MOI 10 at 24 h post infection. The *V. harveyi* strain Vh5 was determined earlier to be the most pathogenic to *Artemia* nauplii (data not shown). Newly hatched *Artemia* nauplii were obtained from the live feed section of IMBBC, HCMR and were disinfected according to the protocol by Gomez-Gil et al. [114]. The *Artemia* nauplii were then washed three times with autoclaved and filtered borehole water (T: 25 °C). Afterwards, 50 *Artemia* nauplii were transferred to each well in Thermo Scientific™ Nunc™ Cell-Culture 6-well plates (Thermo Fisher Scientific 168 Third Avenue, Waltham, MA, USA) with 10 mL autoclaved and filtered borehole water. Aliquots from the washed *Artemia* nauplii were spotted on a bacterial lawn to observe presence of sodium hypochlorite residues. All treatments except for the negative control were inoculated with Vh5 at ~10^−5^ CFU/mL. At 2 h post infection, the single dose vB_VhaM_pir03 treatments were inoculated. For the delayed treatment, inoculation of vB_VhaM_pir03 was only carried out at 24 h post infection. The *Artemia* nauplii were fed with autoclaved *Aeromonas hydrophila* at 10 cells per individuals daily [115]. The sterility of the autoclaved *A. hydrophila* was tested earlier by transferring 10 µL from its suspension to LB and streaking on a LB agar. Individual counts of the *Artemia* nauplii were carried out by visual counting using a NIKON SMZ-800 Stereomicroscope (Nikon Instruments Inc., 1300 Walt Whitman Road Melville, NY 11747-3064, USA) at 24 and 48 h post infection to determine survival percentage. 100 µL of water from each treatment were taken and serially diluted before spotted on TCBS agar at 24 and 48 h post infection to determine the total *Vibrio* load. All treatments were done in triplicates at 25 °C.

### 5.14. Statistical Analysis

One-way ANOVA was performed for the thermal stability and effects of organic solvents assays. Two-way ANOVA was performed for calculation the survival of *Artemia* nauplii (factor A: hours post infection, factor B: treatments) and total *Vibrio* load (factor A: hours post infection, factor B: treatments). Tukey’s HSD post hoc test [116] was used as a multiple comparison tool after ANOVA was performed. Standard error of the mean was displayed for n = 3. All statistical analyses were carried out with PAST: Paleontological Statistics Software Package for Education and Data Analysis version 4.03 [117].

## Figures and Tables

**Table 1:** Host range and efficiency of plating of vB_VhaM_pir03 against selected *Vibrio* spp. On the right is the sensitivity of these strains against antibiotics used in aquaculture.

Efficiency of Plating of vB_VhaM_pir03								Antibiotic Susceptibility Testing						
Species/Strain	Host Range						EOP	Zone of Inhibition Diameter (mm)						
	10^0^	10^−1^	10^−2^	10^−3^	10^−4^	10^−5^	(%)	AMP	TE	OT	OA	FFC	SXT	UB
*Vibrio harveyi*														
*DSM19623	+++	+++	+++	+++	++	+	High	R	S	I	R	S	S	S
SNGR	+++	+++	+++	+++	++	++	High	R	S	R	I	S	R	S
KS6	++	++	++	+	+	-	Low	R	S	R	R	R	R	S
Vh2	+++	+++	++	++	+	-	Medium	R	S	S	R	S	I	S
Vh5	+++	+++	+++	+++	++	+	High	R	S	I	S	S	S	S
VhSernFr	+++	+++	+++	+++	++	+	High	R	S	S	R	I	I	S
VhP1Liv	+++	+++	+++	+++	++	+	High	R	S	I	S	S	S	S
Vhp1Spl	++	+	+	-	-	-	Low							
VhKarx	++	-	-	-	-	-	NF	R	S	I	R	S	S	S
RG1	+++	++	++	+	-	-	Medium	R	S	R	S	S	S	S
Barb A4/1.1	-	-	-	-	-	-	NF	R	S	I	I	S	S	S
SerKid	+++	+++	+++	+++	++	+	Medium	R	S	I	I	S	S	S
SerKid2	+++	+++	+++	+++	++	+	High							
SerSd	+++	++	++	++	+	+	High							
SA 5.1	+++	+++	++	++	++	+	Medium	R	S	I	I	S	R	S
SA 6.1	++	++	+	+	-	-	Low	R	S	I	I	R	R	R
SA 9.2	++	++	++	+	+	+	Medium	R	S	R	I	S	R	I
SA 1.2	++	++	+	+	-	-	Low	R	S	I	R	R	R	R
SA 7.1	+++	+++	++	+	-	-	Low	R	S	S	I	I	R	S
SA 3.1	+++	+++	++	++	++	+	Medium	R	S	I	R	S	R	S
SA 4.1	+++	+	+	+	-	-	Low	R	S	I	I	R	R	R
SA 2.1	++	+	+	+	-	-	Low	R	S	R	R	S	S	S
Vh No. 22	++	++	++	+	-	-	Low	R	S	I	I	I	S	I
Vh6	-	-	-	-	-	-	NF	R	S	I	I	S	I	S
*Vibrio alginolyticus*														
V1	+++	+++	+++	+++	++	+	High	R	S	I	I	S	S	S
V2	+++	+++	++	++	+	-	Low	R	S	I	I	S	S	S
HCMR 1 Art. 3	++	++	++	++	+	-	High	R	S	S	I	S	R	S
*Vibrio campbellii*														
VIB391	+++	+++	+++	++	-	-	Medium	R	S	I	I	S	S	S
*Vibrio owensii*														
SA 1.1	++	++	++	++	+	+	Medium	R	S	I	R	R	R	R
SA 9.1	++	+	+	+	-	-	Low	R	S	R	I	R	R	S
Other *Vibrio* spp.														
Art. 2	+++	+++	++	++	++	+	High	R	S	R	I	R	R	R
Rot. 2	+++	++	-	-	-	-	Low	R	S	R	S	R	R	I
Barb A4/1.2	+++	+++	++	++	+	-	High	R	S	I	I	S	S	S
Rot. Vib. 5	+++	+++	+++	++	-	-	Medium	R	S	S	I	R	R	S

Abbreviations: EOP, efficiency of plating; AST, antibiotic susceptibility testing; AMP, ampicillin; TE, tetracycline; OT, oxytetracycline; OA, oxalinic acid; FFC, florfenicol; SXT, sulfamethoxazole/trimethoprim; UB, flumequine. Efficiency of plating: NF, no formation; +++, single large clearing zone; ++, ≥30 small plaques; +, <30 small plaques; NF, no formation; High, EOP > 10.0%; medium, 0.5% < EOP < 9.9%; low, EOP < 0.5%. *DSM19623 is used as the reference strain for efficiency of plating (EOP) calculations. Antibiotic susceptibility testing: R, resistant; I, intermediate; S, susceptible2.3. Stability of vB_VhaM_pir03 in different temperatures and organic solvents.

**Table 2:** Summary table of vB_VhaM_pir03 ORFs that were annotated with relevant information based on significant amino acid sequence and protein structural homologies (E-value ≤ 10^−3^).

	Predicted Functions	Start	End	Length	Direction	NCBI BLASTP Best Hit	E-Value	Similarity Score (%)	Gene Onthology	InterPro	NCBI CDD Best Hit	E-Value	HHPRED Best Hit	E-Value	Probability (%)
ORF 2	Baseplate structural protein	3242	6268	3027	Reverse	*Vibrio* phage BONAISHI AXH71039.1	1.05 × 10^−15^	42.48			cl33689|long tail fiber, proximal subunit	5.51 × 10^−3^	1S2E_B|Bacteriophage T4	1.20 × 10^−5^	98.20
ORF 3	Hypothetical protein	6278	7180	903	Reverse	*Vibrio* phage BONAISHI AXH71040.1	2.0 × 10^−40^	57.70							
ORF 4	Putative tail protein	7192	8181	990	Reverse	*Vibrio* phage BONAISHI AXH71041.1	5.39 × 10^−72^	56.00							
ORF 6	Hypothetical protein	8315	9526	1212	Forward	*Vibrio* phage vB_VmeM-Yong XC31 QAX96157.1	3.0 × 10^−30^	47.20							
ORF 7	ABC-type ATPase	9568	12,276	2709	Reverse	*Vibrio* phage vB_VmeM-Yong XC31 QAX96150.1	1.76 × 10^−109^	50.20			cd00267|ATP-binding cassette transporter nucleotide-binding domain	1.30 × 10^−7^	6S6V_D|*Escherichia coli*	4.40 × 10^−23^	99.96
ORF 8	Membrane-puncturing device	12,113	12,937	825	Forward	*Vibrio* phage BONAISHI AXH70744.1	2.51 × 10^−66^	61.50					6ORJ_A|*Pseudomonas* virus phiKZ	4.60 × 10^−89^	100.00
ORF 9	Hypothetical protein	12,947	13,567	621	Forward	*Vibrio* phage BONAISHI AXH70745.1	1.43 × 10^−10^	56.31							
ORF 10	PAAR-repeat containing protein	13,560	13,859	300	Forward	*Vibrio* phage BONAISHI AXH70746.1	1.30 × 10^−21^	69.40		IPR008727	cd14737|proline-alanine-alanine-arginine (PAAR) domain	1.46 × 10^−29^	4KU0_D|Bacteriophage T4	2.70 × 10^−11^	99.40
ORF 11	PAAR-motif protein	13,718	13,891	174	Forward	*Vibrio* phage 03O._10N.2646.F8] AUR83144.1	9.00 × 10^−3^	6.64							
ORF 12	Hypothetical protein	13,901	14,512	612	Reverse	*Vibrio* phage vB_VmeM-Yong XC31 QAX96156.1	4.76 × 10^−31^	54.80							
ORF 13	Hypothetical protein	14,523	158,181	1296	Reverse	*Vibrio* phage vB_VmeM-Yong XC31 QAX96155.1	8.43 × 10^−68^	53.60							
ORF 16	Ribonuclease HI (EC 3.1.26.4) CDS	16,667	18,028	1362	Forward	*Vibrio* phage BONAISHI AXH70751.1	1.35 × 10^−59^	48.00	F: GO:0016787	IPR036397	cd09278|RNase HI family found mainly in prokaryotes	1.40 × 10^−36^	4MH8_A|Moloney murine leukemia virus	2.90 × 10^−9^	99.07
ORF 17	Hypothetical protein	18,081	18,920	840	Reverse	*Vibrio* phage BONAISHI AXH70752.1	8.97 × 10^−21^	50.70							
ORF 18	UvsX protein	18,757	20,484	1728	Forward	*Vibrio* phage BONAISHI AXH70753.1	3.12 × 10^−171^	67.50					3IO5_B|Bacteriophage T4.	4.60 × 10^−26^	99.95
ORF 20	DNA-directed RNA polymerase	20,726	21,496	771	Forward	*Vibrio* phage BONAISHI AXH70756.1	2.85 × 10^−61^	66.70					2A6H_M|*Thermus thermophilus*	3.80 × 10^−32^	99.98
ORF 21	Hypothetical protein	21,489	21,896	408	Forward	*Vibrio* phage BONAISHI AXH70757.1	7.61 × 10^−7^	51.18							
ORF 22	Hypothetical protein	21,889	22,572	684	Forward	*Vibrio* phage BONAISHI AXH70758.1	3.23 × 10^−23^	49.10							
ORF 25	Hypothetical protein	23,638	24,297	660	Forward	*Vibrio* phage BONAISHI AXH70761.1	2.49 × 10^−42^	58.30							
ORF 26	Hypothetical protein	24,395	25,489	1095	Forward	*Vibrio* phage BONAISHI AXH70762.1	1.87 × 10^−9^	40.40							
ORF 27	Hypothetical protein	25,529	27,553	2025	Reverse	*Vibrio* phage BONAISHI AXH70763.1	4.37 × 10^−162^	59.50							
ORF 28	Glycoside hydrolase	27,596	33,355	5760	Reverse	*Vibrio* phage BONAISHI AXH70764.1	6.29 × 10^−81^	52.30			pfam01551|Peptidase family M23	1.40 × 10^−35^	4RNZ_A|*Helicobacter pylori*	4.70 × 10^−15^	99.57
ORF 29	DNA-directed RNA polymerase subunit alpha	33,458	34,960	1503	Forward	*Vibrio* phage BONAISHI AXH70765.1	1.65 × 10^−173^	74.10					5ZX3_D|*Mycobacterium tuberculosis*	2.00 × 10^−49^	100.00
ORF 30	HNH endonuclease	35,019	35,972	954	Forward	*Chryseobacterium gleum*] WP_002984461.1	5.39 × 10^−13^	57.30	F: GO:0004519	IPR010896	pfam13392|HNH endonuclease	1.45 × 10^−10^	1U3E_M|*Bacillus* phage SPO1	4.90 × 10^−29^	99.97
ORF 33	Hypothetical protein	36,322	36,495	174	Forward	*Vibrio* phage vB_VmeM-Yong XC31 QAX96130.1	6.44 × 10^−10^	70.90							
ORF 34	RNA-polymerase beta subunit	36,504	37,889	1386	Forward	*Vibrio* phage BONAISHI AXH70766.1	7.28 × 10^−71^	55.40							
ORF 35	Homing endonuclease	37,939	39,027	1089	Forward	*Vibrio* phage vB_VmeM-Yong XC32 QAX96446.1	2.5 × 10^−101^	61.50					3R3P_A*|Bacillus* phage 0305phi8-36	1.70 × 10^−3^	97.46
ORF 38	DNA-directed RNA polymerase subunit alpha	39,437	42,001	2565	Forward	*Vibrio* phage vB_VmeM-Yong XC31 QAX96126.1	0.0 × 10^0^	64.00	F: GO:0003899	SSF64484			6J9E_C|*Xanthomonas oryzae* (strain PXO99A)	1.50 × 10^−77^	100.00
ORF 40	Hypothetical protein	42,278	43,774	1497	Forward	*Vibrio* phage BONAISHI AXH70769.1	4.42 × 10^−38^	46.31							
ORF 41	Hypothetical protein	43,830	44,558	729	Forward	*Vibrio* phage BONAISHI AXH70770.1	1.11 × 10^−65^	71.50							
ORF 42	Prohead core protein protease	44,567	45,388	822	Forward	*Vibrio* phage vB_VmeM-Yong XC31 QAX96122.1	1.91 × 10^−37^	50.40					5JBL_E|Bacteriophage T4	2.20 × 10^−25^	99.93
ORF 51	Tail-tube protein	48,267	49,133	867	Reverse	*Vibrio* phage vB_VmeM-Yong XC31 QAX96106.1	4.32 × 10^−61^	59.70					5IV5_DE|Bacteriophage T4	5.80 × 10^−2^	96.67
ORF 52	Tail sheath protein	49,188	51,218	2031	Reverse	*Vibrio* phage BONAISHI AXH70778.1	6.24 × 10^−162^	59.70					3SPE_A|*Pseudomonas* phage phiKZ	2.40 × 10^−69^	100.00
ORF 54	Hypothetical protein	51,437	52,378	942	Forward	*Vibrio* phage vB_VmeM-Yong XC31 QAX96104.1	8.14 × 10^−19^	48.10							
ORF 55	Virion structural protein	52,341	54,992	2652	Forward	*Vibrio* phage BONAISHI AXH70780.1	1.42 × 10^−101^	48.90							
ORF 56	Hypothetical protein	55,004	56,665	1662	Forward	*Vibrio* phage BONAISHI AXH70781.1	1.49 × 10^−120^	58.20							
ORF 57	Phage DNA helicase or terminase, large subunit	56,711	58,900	2190	Forward	*Vibrio* phage BONAISHI AXH70782.1	0.0 × 10^0^	69.50					3CPE_A|Bacteriophage T4	2.60 × 10^−34^	100.00
ORF 58	Hypothetical protein	58,943	60,159	1217	Reverse	*Vibrio* phage BONAISHI AXH70784.1	3.34 × 10^−110^	65.90							
ORF 60	Hypothetical protein	60,314	61,015	702	Forward	*Vibrio* phage BONAISHI AXH70786.1	1.02 × 10^−8^	48.60							
ORF 62	Hypothetical protein	61,415	62,446	1032	Forward	*Vibrio* phage BONAISHI AXH70788.1	2.60 × 10^−46^	60.50							
ORF 64	RNA-binding protein	62,625	64,175	1551	Reverse	*Vibrio* phage BONAISHI AXH70790.1	6.79 × 10^−5^	44.30	F: GO:0003723	IPR037214			2NVO_A|*Deinococcus radiodurans*	1.60 × 10^−62^	100.00
ORF 90	Hypothetical protein	69,563	70,327	765	Reverse	*Vibrio* phage BONAISHI AXH70799.1	5.98 × 10^−37^	58.00							
ORF 99	Hypothetical protein	74,169	74,585	417	Forward	*Vibrio* phage BONAISHI AXH70809.1	2.55 × 10^−10^	51.20							
ORF 101	DNA polymerase	74,767	76,938	2172	Forward	*Vibrio* phage BONAISHI AXH70810.1	0.0 × 10^0^	64.80	F: GO:0000166	IPR006134	cl33389|DNA polymerase type-B family	4.62 × 10^−3^	3QEX_A|Bacteriophage RB69	2.60 × 10^−28^	99.97
ORF 102	Hypothetical protein	76,983	78,047	1065	Reverse	*Vibrio* phage BONAISHI AXH70811.1	1.42 × 10^−36^	52.10							
ORF 105	Hypothetical protein	78,386	80,323	1938	Forward	*Vibrio* phage BONAISHI AXH70812.1	7.30 × 10^−68^	47.20							
ORF 106	DNA-directed RNA polymerase	80,416	81,771	1356	Forward	*Vibrio* phage vB_VmeM-Yong XC31 QAX96059.1	8.5 × 10^−130^	67.60	F: GO:0003677	SSF64484	cl37096|DNA-directed RNA polymerase, beta subunit	1.24 × 10^−3^	6RFL_A|*Vaccinia* virus GLV-1h68	1.30 × 10^−36^	100.00
ORF 107	HNH endonuclease	81,598	82,680	1083	Forward	*Pseudomonas* sp. JY-Q WP_064614171.1	1.0 × 10^−9^	45.21							
ORF 109	RNA polymerase beta prime subunit	82,888	83,265	378	Forward	*Vibrio* phage BONAISHI AXH70813.1	6.86 × 10^−8^	53.10							
ORF 112	Hypothetical protein	84,239	84,934	606	Forward	*Vibrio* phage vB_VmeM-Yong XC31 QAX96047.1	2.32 × 10^−17^	49.00							
ORF 113	Hypothetical protein	84,968	85,369	402	Forward	*Vibrio* phage BONAISHI AXH70815.1	5.83 × 10^−20^	54.50							
ORF 114	Hypothetical protein	85,371	85,847	477	Forward	*Vibrio* phage BONAISHI AXH70816.1	3.08 × 10^−18^	59.20							
ORF 115	Putative nuclease SbcD subunit D	85,759	86,961	1203	Forward	*Vibrio* phage BONAISHI AXH70817.1	1.88 × 10^−74^	56.10	F: GO:0016787	IPR029052	cl33866|DNA repair exonuclease SbcCD nuclease subunit	1.92 × 10^−9^	6S6V_B|*Escherichia coli*	4.30 × 10^−30^	100.00
ORF 116	Hypothetical protein	86,958	87,755	798	Forward	*Vibrio* phage vB_VmeM-Yong XC31 QAX96043.1	6.97 × 10^−44^	55.00							
ORF 117	Hypothetical protein	87,818	88,516	699	Forward	*Vibrio* phage BONAISHI AXH70819.1	7.16 × 10^−35^	55.40							
ORF 118	Hypothetical protein	88,692	90,155	1464	Forward	*Vibrio* phage vB_VmeM-Yong XC31 QAX96041.1	2.29 × 10^−68^	52.10							
ORF 119	Hypothetical protein	90,170	91,627	1458	Forward	*Vibrio* phage BONAISHI AXH70821.1	1.49 × 10^−67^	50.00							
ORF 120	Hypothetical protein	91,671	93,587	1917	Forward	*Vibrio* phage BONAISHI AXH70822.1	7.01 × 10^−27^	49.00							
ORF 122	RNA polymerase beta subunit	93,898	96,105	2208	Forward	*Vibrio* phage BONAISHI AXH70824.1	1.04 × 10^−152^	55.90	F: GO:0003677	IPR007120	cl37028|DNA-directed RNA polymerase, beta subunit.	2.01 × 10^−8^	6PST_I|*Escherichia coli*	1.00 × 10^79^	100.00
ORF 123	RNA polymerase beta subunit	96,116	98,080	1965	Forward	*Vibrio* phage vB_VmeM-Yong XC31 QAX96036.1	0.00 × 10^0^	60.40	F: GO:0003899				6PST_J|*Escherichia coli*	1.30 × 10^−35^	100.00
ORF 125	ATP-dependent DNA helicase uvsW	98,305	99,693	1389	Forward	*Vibrio* phage vB_VmeM-Yong XC31 QAX96032.1	1.91 × 10^−153^	68.70			cl34083|Superfamily II DNA or RNA helicase	1.64 × 10^−12^	2OCA_A| Bacteriophage T4	9.20 × 10^−32^	100.00
ORF 127	ATP-dependent Clp protease proteolytic subunit	100,236	100,730	495	Forward	*Vibrio* phage BONAISHI AXH70828.1	9.08 × 10^−26^	54.00	F: GO:0004252	IPR001907	cl23717|Crotonase/Enoyl-Coenzyme A (CoA) hydratase superfamily	5.81 × 10^−25^	2FZS_H|*Escherichia coli*	1.20 × 10^−22^	99.93
ORF 129	Hypothetical protein	101,203	102,672	1470	Reverse	*Vibrio* phage BONAISHI AXH70830.1	1.17 × 10^−14^	44.60							
ORF 132	Hypothetical protein	103,126	103,947	822	Forward	*Vibrio* phage BONAISHI AXH70832.1	7.46 × 10^−41^	55.60							
ORF 133	RNA polymerase beta prime subunit	103,982	105,211	1230	Forward	*Vibrio* phage BONAISHI AXH70833.1	2.45 × 10^−123^	66.00	F: GO:0003899		cl32391|DNA-directed RNA polymerase subunit beta	1.46 × 10^−8^	6PST_J|*Escherichia coli*	7.20 × 10^−46^	100.00
ORF 134	Putative replication protein A family	105,237	105,911	675							cl09930|Replication protein A, class 2b aminoacyl-tRNA synthetases				
ORF 137	Hypothetical protein	106,600	107,946	1347	Forward	*Vibrio* phage BONAISHI AXH70836.1	5.35 × 10^−5^	45.90							
ORF 138	DNA polymerase	107,997	109,724	1728	Reverse	*Vibrio* phage BONAISHI AXH70837.1	0.00 × 10^0^	74.50	F: GO:0003676	IPR036397	smart00486|DNA polymerase type-B family	5.325 × 10^−8^	3QEX_A|Bacteriophage RB69	6.40 × 10^−44^	100.00
ORF 139	Virion structural protein	109,804	111,066	1263	Forward	*Vibrio* phage BONAISHI AXH70838.1	1.04 × 10^−36^	48.90	F: GO:0004222				6AIT_C|*Escherichia coli* (strain K12)	3.30 × 10^−6^	99.05
ORF 140	Hypothetical protein	111,070	113,046	1977	Forward	*Vibrio* phage BONAISHI AXH70839.1	4.21 × 10^−9^	42.80							
ORF 141	Virion structural protein	113,085	115,931	2847	Reverse	*Vibrio* phage BONAISHI AXH70840.1	1.42 × 10^−178^	56.70							
ORF 142	Virion structural protein	115,924	116,970	1047	Reverse	*Vibrio* phage BONAISHI AXH70841.1	1.32 × 10^−95^	60.60							
ORF 143	Capsid protein	117,030	118,136	1107	Forward	*Vibrio* phage BONAISHI AXH70842.1	1.63 × 10^−68^	58.60							
ORF 144	Virion structural protein	118,147	119,028	882	Forward	*Vibrio* phage BONAISHI AXH70843.1	7.55 × 10^−24^	50.00							
ORF 146	Hypothetical protein	119,612	120,913	1302	Forward	*Vibrio* phage BONAISHI AXH70845.1	2.53 × 10^−6^	43.20							
ORF 148	Putative internal head protein	122,313	123,377	1245	Forward						cl20461|phiKZ-like phage internal head proteins	1.32 × 10^−3^	.		
ORF 149	Hypothetical protein	123,449	125,053	1605	Forward	*Vibrio* phage BONAISHI AXH70848.1	2.17 × 10^−108^	56.10							
ORF 150	Virion structural protein	125,053	126,270	1218	Forward	*Vibrio* phage BONAISHI AXH70849.1	1.52 × 10^−65^	55.20							
ORF 152	Virion structural protein	126,970	128,334	1365	Forward	*Vibrio* phage BONAISHI AXH70851.1	1.52 × 10^−46^	50.80							
ORF 153	DNA helicase	128,374	129,921	1548	Reverse	*Vibrio* phage BONAISHI AXH70852.1	2.72 × 10^−177^	70.10	F: GO:0003678				6BBM_A|*Escherichia coli* O111	1.50 × 10^−36^	100.00
ORF 156	Major capsid protein	130,604	132,769	2166	Forward	*Vibrio* phage BONAISHI AXH70854.1	3.91 × 10^−147^	63.10							
ORF 159	Hypothetical protein	134,018	135,589	1572	Forward	*Vibrio* phage BONAISHI AXH70856.1	9.35 × 10^−141^	61.50							
ORF 162	DUF723 domain-containing protein	138,774	139,013	240	Forward	*Vibrio mediterranei* WP_096444327.1	5.15 × 10^−12^	61.90	F: GO:0004519						
ORF 163	Endonuclease	139,080	139,541	462	Forward	*Vibrio* phage 1.225.O._10N.261.48.B7 AUR96455.1	2.54 × 10^−7^	46.00					6SEI_A|*Thielavia terrestris*	1.80 × 10^−4^	97.77
ORF 164	Holliday junction resolvase	139,578	140,171	594	Reverse	*Vibrio* phage BONAISHI AXH70859.1	8.65 × 10^−41^	62.90	P: GO:0009987	IPR036397	cl21482|Crossover junction endodeoxyribonuclease RuvC and similar proteins	1.05 × 10^−3^	6LW3_B|*Escherichia coli*	4.70 × 10^−22^	99.91
ORF 165	Virion structural protein	140,171	141,001	831	Reverse	*Vibrio* phage BONAISHI AXH70860.1	2.26 × 10^−69^	60.40							
ORF 166	Virion structural protein	141,013	143,070	2058	Reverse	*Vibrio* phage BONAISHI AXH70861.1	1.93 × 10^−125^	56.50							
ORF 167	Putative portal protein	143,175	145,940	2766	Forward	*Vibrio* phage BONAISHI AXH70862.1	3.34 × 10^−132^	55.60			cl27451|Hypothetical protein	3.55 × 10^−6^	3JA7_I|Bacteriophage T4	2.60 × 10^−5^	98.45
ORF 168	Putative hydrolase	145,952	146,905	954	Forward	*Vibrio* phage BONAISHI AXH70863.1	9.39 × 10^−31^	50.20	C: GO:0016020				4F55_A|*Bacillus cereus*	1.10 × 10^−24^	99.92
ORF 177	Hypothetical protein	150,443	151,204	762	Forward	*Vibrio* phage vB_VhaS-a ANO57550.1	1.74 × 10^−30^	57.50							
ORF 178	Hypothetical protein	151,281	151,952	672	Forward	*Vibrio* phage vB_VhaS-a ANO57549.1	9.91 × 10^−40^	61.50							
ORF 182	Glycohydrolase	154,582	155,496	915	Forward	*Vibrio* phage BONAISHI AXH70879.1	2.61 × 10^−62^	54.81	F: GO:0016787	IPR002477					
ORF 183	Transcription factor: type II DNA-Binding	155,609	156,363	855									1WTU_A|*Bacillus* phage SPO1	6.60 × 10^−3^	97.04
ORF 184	Transcription factor: type II DNA-Binding	156,379	156,868	510									1WTU_A|*Bacillus* phage SPO1	6.60 × 10^−3^	97.04
ORF 185	Hypothetical protein	156,896	157,618	723	Forward	*Vibrio* phage BONAISHI AXH70881.1	6.55 × 10^−12^	47.83							
ORF 193	Putative DNA repair exonuclease	161,416	162,201	786	Forward	*Vibrio* phage pVa-21 AQT28114.1	1.92 × 10^−20^	53.60	F: GO:0004527	IPR036412			5UJ0_A|Bacteriophage T4	7.70 × 10^−11^	99.26
ORF 194	Nucleotide binding protein	162,149	162,760	612	Forward	*Vibrio* phage BONAISHI AXH70900.1	9.48 × 10^−16^	54.40			cl17018|Fanconi anemia ID complex proteins FANCI and FANCD2	7.57 × 10^−3^	3GH1_B|*Vibrio cholerae* O1 biovar El Tor str. N16961	5.20 × 10^−7^	98.66
ORF 196	Hypothetical protein	163,750	164,421	672	Forward	*Vibrio* phage vB_VmeM-Yong XC31 QAX96244.1	2.14 × 10^−100^	81.60							
ORF 197	Hypothetical protein	164,476	164,808	333	Forward	*Vibrio* phage vB_VmeM-Yong XC31 QAX96240.1|	1.00 × 10^−3^	38.89							
ORF 198	DEAD-like helicase	164,871	166,841	1971	Forward	*Vibrio* phage vB_VmeM-Yong XC31 QAX96243.1	1.70 × 10^−166^	60.90	P:GO:0000733	IPR014001	cd18793|C-terminal helicase domain of the SNF family helicases.	1.80 × 10^−16^	2OCA_A|Bacteriophage T4	1.10 × 10^−29^	99.98
ORF 199	EAR-like protein	166,948	168,591	1647						IPR009039					
ORF 200	Putative Palindromic Amphipathic Repeat Coding Elements (PARCEL)	168,504	187,867	19,254						IPR011889	pfam03382|Mycoplasma protein of unknown function, DUF285	1.29 × 10^−44^			
ORF 202	DNA-packaging protein: hydrolase	188,001	188,966	966	Forward	*Pseudomonas* virus phiKZ NP_803591.1	5.69 × 10^−20^	53.10	C: GO:0016020				2O0J_A|Bacteriophage T4	1.80 × 10^−24^	99.92
ORF 207	UV-endonuclease	191,809	192,780	972	Forward	*Vibrio* phage 1.084.O._10N.261.49.F5 AUR86431.1	6.23 × 10^−67^	55.90	F: GO:0004519	IPR004601	cl23721|AP endonuclease family 2	2.84 × 10^−48^	3TC3_B|*Sulfolobus acidocaldarius*	1.20 × 10^−26^	99.96
ORF 209	Dihydrofolate reductase (EC 1.5.1.3) CDS	193,421	194,152	732	Forward	*Meiothermus ruber* HFG20084.1	2.1 × 10^−22^	59.30	P: GO:0008152	IPR001796	cd00209|Dihydrofolate reductase (DHFR)	9.07 × 10^−39^	1JUV_A|Bacteriophage T4	1.50 × 10^−20^	99.87
ORF 223	Putative nucleotidyl transferase	202,749	203,570	822	Forward	*Yersinia* phage phiR1-37 YP_004934311.1	3.51 × 10^−13^	51.80	F: GO:0016740	IPR043519	cl35051|elongation factor Tu	3.27 × 10^−4^	2FCL_A|*Thermotoga maritima*	6.70 × 10^−12^	99.44
ORF 225	Putative N-acetyltransferase	204,006	204,428	423						IPR016181			5Z6N_A| *Escherichia coli* (strain K12)		
ORF 229	Putative HTH-type transcription I regulator MqsA	205,857	506,309	453									3GA8_A|*Escherichia coli* K-12		
ORF 230	Hypothetical protein VPIG_00040	206,325	206,852	528	Forward	*Vibrio* phage PWH3a-P1 YP_007675900.1	5.86 × 10^−9^	56.40							
ORF 231	Hypothetical protein SAMN05421742_1266	206,849	207,121	453	Forward	*Roseospirillum parvum* SDH93001.1|	1.94 × 10^−5^	54.70							
ORF 243	Putative glycosylhydrolase	214,545	215,822	1278						IPR013320					
ORF 245	Hypothetical protein BCS93_11070	217,217	218,539	1323	Reverse	*Vibrio breoganii* PMP10208.1	8.63 × 10^−13^	59.50					3ZYP_A|*Hypocrea jecorina*	6.00 × 10^−3^	97.33
ORF 247	Hypothetical protein	218,552	219,949	1398	Reverse	*Vibrio breoganii* WP_133150968.1	1.32 × 10^−10^	42.50					3ZYP_A|*Hypocrea jecorina*	8.80 × 10^−4^	97.72
ORF 248	Hypothetical protein NVP1169O_83	221,333	222,682	1350	Reverse	*Vibrio* phage 1.169.O._10N.261.52.B1 AUR92111.1	5.68 × 10^−10^	40.40							
ORF 249	Hypothetical protein BDU10_8600	222,682	223,677	996	Reverse	*Burkholderia* sp. CF145 OYD65949.1	9.15 × 10^−15^	53.10							
ORF 250	Polynucleotide kinase	223,801	224,589	789	Forward	*Aeromonas* virus Aeh1 NP_943967.1	1.30 × 10^−11^	37.40			cl40282|HAD domain in Swiss Army Knife RNA repair proteins.	1.12 × 10^−8^	5UJ0_A|Bacteriophage T4	1.80 × 10^−4^	98.02
ORF 256	SH3 protein	228,286	228,981	695							cl17036|Src Homology 3 domain superfamily	6.19 × 10^−3^			
ORF 262	Phosphagen kinase	232,254	232,973	720	Forward						cl02823|Phosphagen (guanidino) kinases				
ORF 263	DNA polymerase accessory protein 44: AAA+, ATP hydrolase	233,047	234,390	1344	Forward	*Salmonella enterica* EAZ2022740.1	2.56 × 10^−30^	50.70	F: GO:0000166	IPR003593			3U61_D|Bacteriophage T4}	9.00 × 10^−12^	99.45
ORF 267	Ribonuclease E/G	236,230	236,958	729							cl29166|Ribonuclease E/G family	1.32 × 10^−3^			
ORF 272	DNA polymerase II large subunit	239,038	239,373	336							cl36419|DNA-directed DNA polymerase II large subunit	3.90 × 10^−3^			
ORF 275	NAD-dependent DNA ligase LigA	240,880	242,817	1938	Forward	*Salinivibrio* sp. ES.052 WP_074213176.1	2.87 × 10^−131^	57.20	P: GO:0006259	IPR001357	cl35633|NAD-dependent DNA ligase LigA; Validated	0.00 × 10^0^	5TT5_A|*Escherichia coli* K12	1.50 × 10^−121^	100.00
ORF 278	GTP cyclohydrolase II	244,297	244,800	504	Forward	*Vibrio* phage PWH3a-P1 YP_007676007.1	2.19 × 10^−30^	61.60							
ORF 281	Thymiylate kinase	245,594	246,316	723	Forward	*Firmicutes bacterium* CAG:582 CDB28696.1	3.93 × 10^−34^	54.80	P: GO:0006796.	IPR039430	cl17190|Nucleoside/nucleotide kinase (NK).	1.10 × 10^−26^	3LV8_A|*Vibrio cholerae* O1 biovar El Tor	2.50 × 10^−22^	99.92
ORF 283	Ribonucleotide reductase of class Ia (aerobic), alpha subunit	246,875	249,163	2289	Forward	*Rodentibacter pneumotropicus* WP_077664105.1	0.00 × 10^0^	71.30	F: GO:0000166	IPR005144	cl32350|ribonucleoside-diphosphate reductase subunit alpha.	0.00 × 10^0^	2XAP_A|*Escherichia coli*	5.10 × 10^−23^	100.00
ORF 286	Ribonucleotide reductase of class Ia (aerobic), beta subunit	250,213	251,331	1119	Forward	*Sulfurivirga caldicuralii* WP_074201546.1	8.00 × 10^−139^	69.40	F: GO:0004748		cl00264|Ferritin-like superfamily of diiron-containing four-helix-bundle proteins	0.00 × 10^0^	1MXR_B|*Escherichia coli*	1.90 × 10^−56^	100.00
ORF 287	BspA family leucine-rich repeat surface protein	251,408	255,091	3684	Forward	*Helicobacter bizzozeronii* WP_158656920.1	1.25 × 10^−36^	46.00	C: GO:0016020	IPR005046	cl37689|Mycoplasma protein of unknown function, DUF285	2.51 × 10^−27^			
ORF 299	PIN terminus	261,891	262,838	978						IPR002716	cl28905|PIN (PilT N terminus) domain: Superfamily	2.33 × 10^−6^	2HWY_A|*Homo sapiens*	4.10 × 10^−3^	96.35
ORF 308	Hypothetical protein	266,214	266,954	741	Forward	*Cellulomonas aerilata* WP_146903668.1	2.66 × 10^−4^	41.20					6HIY_DS|*Trypanosoma brucei brucei*	5.50 × 10^−11^	99.13
ORF 310	Glutaredoxin	267,350	268,000	651							cl35908|glutaredoxin 2	2.14 × 10^−3^			
ORF 316	Asp/Glu/Hydantoin racemase	270,693	271,877	1185							cl00518|Asp/Glu/Hydantoin racemase	5.48 × 10^−3^			
ORF 319	RNA-binding protein	274,073	274,750	678	Forward	*Vibrio* phage BONAISHI AXH70995.1	4.95 × 10^−20^	51.30	F: GO:0016787	IPR036397	cl10012|DnaQ-like (or DEDD) 3′-5′ exonuclease domain superfamily	1.01 × 10^−13^	6N6A_A|*Vibrio cholerae*	1.60 × 10^−15^	99.70
ORF 323	Thymidylate synthase (EC 2.1.1.45)	276,131	277,057	927	Forward	*Vibrio* phage 2.275.O._10N.286.54.E11 AUS02985.1	3.26 × 10^−76^	62.60	P: GO:0008152	IPR023451	cl19097|Thymidylate synthase and pyrimidine hydroxy methylase.		1TIS_A|Bacteriophage T4		
ORF 326	Nucleoside Triphosphate Pyrophosphohydrolase	277,926	278,654	729	Forward	*Vibrio* phage vB_VmeM-Yong XC31 QAX96185.1	4.77 × 10^−10^	58.40			cl16941|Nucleoside Triphosphate Pyrophosphohydrolase (EC 3.6.8) MazG-like domain superfamily	4.07 × 10^−3^	2YF4_B|*Deinococcus radiodurans*	8.80 × 10^−20^	99.83
ORF 329	Hypothetical protein	279,832	280,650	819	Forward					IPR006530					
ORF 330	Hypothetical protein	280,703	281,116	414	Reverse	*Vibrio* phage BONAISHI AXH71034.1	1.91 × 10^−13^	54.40							
ORF 331	Hypothetical protein yiiX	281,272	281,703	432	Reverse	*Vibrio* phage vB_VmeM-Yong XC31 QAX96165.1	2.49 × 10^−27^	55.60		IPR038765	cl21534|NlpC/P60 family.	4.98 × 10^−3^	2IF6_A|*Escherichia coli*	4.10 × 10^−21^	99.87
ORF 332	Hypothetical protein	281,713	282,711	999	Reverse	*Vibrio* phage BONAISHI AXH71036.1	1.57 × 10^−41^	47.40							

**Table 3:** List of bacterial strains used in this study with their methods of identification and location of isolation.

Bacterial Strains	Method of Identification	Location
*Vibrio harveyi*		
DSM19623	ENA Accession No: BAOD01000001	USA
SNGR	BIOLOG GENiii	Greece
KS6	BIOLOG GENiii	Greece
Vh2	BIOLOG GENiii, toxR (+)	Greece
Vh5	BIOLOG GENiii, toxR (+)	Greece
VhSernFr	BIOLOG GENiii, toxR (+)	Greece
VhP1 Liv	toxR (+)	Greece
VhP1 Spl	toxR (+)	Greece
VhKarx	BIOLOG GENiii, toxR (+)	Greece
RG1	toxR (+)	Greece
Barb A4/1.1	BIOLOG GENiii, toxR (+)	Greece
SerKid	BIOLOG GENiii	Greece
SerKid2	BIOLOG GENiii	Greece
SerSd	BIOLOG GENiii	Greece
SA 5.1	16S rRNA	KSA
SA 6.1	16S rRNA	KSA
SA 9.2	16S rRNA	KSA
SA 1.2	16S rRNA	KSA
SA 7.1	16S rRNA	KSA
SA 3.1	16S rRNA	KSA
SA 4.1	16S rRNA	KSA
SA 2.1	16S rRNA	KSA
Epith. D	BIOLOG GENiii	Greece
Vh No. 22	BIOLOG GENiii	Greece
Vh6	BIOLOG GENiii	Greece
*Vibrio alginolyticus*		
V1	BIOLOG GENiii	Greece
V2	BIOLOG GENiii	Greece
HCMR 1 Art. 3	Clinical strain	Greece
DSM 2171	ENA Accession No.: AB372523	Japan
Valg HCMR	BIOLOG GENiii	Greece
Skironis-2	BIOLOG GENiii	Greece
NS A6	BIOLOG GENiii	Greece
Rot. Vib. 5	BIOLOG GENiii	Greece
*Vibrio anguillarum*		
90-11-286	Clinical strain	Denmark
VIB 44	Clinical strain	Italy
VIB 64	Clinical strain	Spain
VIB 243	Clinical strain	USA
*Vibrio campbellii*		
VIB391	NCBI RefSeq No: GCF_002078065.1	Thailand
*Vibrio owensii*		
SA 1.1	16S rRNA	KSA
SA 9.1	16S rRNA	KSA
*Vibrio parahaemolyticus*		
VPINH	BIOLOG GENiii	Greece
*Vibrio splendidus*		
Barb A4/2	BIOLOG GENiii	Greece
VaAn	Clinical strain	Greece
Other *Vibrio* spp.		
Art. 1	TCBS	Greece
Art. 2	TCBS	Greece
Rot. 2	toxR (+)	Greece
Barb A4/1.2	TCBS	Greece
Rot. Vib. 1	TCBS	Greece
Rot. Vib. 2	TCBS	Greece
Rot. Vib. 3	TCBS	Greece
Rot. Vib. 4	TCBS	Greece
Rot. Vib. 6	TCBS	Greece

Abbreviations: TCBS, thiosulfate-citrate-bile salts; ENA, European Nucleotide Archive; NCBI, National Center of Biotechnology Information; KSA, Kingdom of Saudi Arabia.

**Table 4:** Disk diffusion interpretive criteria for antibiotic susceptibility testing.

Antimicrobial Agent	Disk Diffusion (μg)	Zone Diameter (mm) Interpretive Criteria		
		S	I	R
Ampicillin	10	≥17	14–16	≤13
Tetracycline	30	≥15	12–14	≤11
Sulphamethoxazole/trimethoprim	25	≥16	11–15	≤10
Oxytetracycline ^a^	30	≥27	17–26	≤16
Florfenicol ^b^	30	≥18	13–17	≤12
Oxalinic acid ^c^	2	≥19	14–18	≤13
Flumequine ^c^	30	≥19	14–18	≤13

Abbreviations: S, susceptible; I, medium; R, resistant. ^a^ based on oxytetracycline breakpoint established by Uhland and Higgins (2006). ^b^ based on analogues in [88] clinical breakpoints for *Vibrio* spp. including *Vibrio cholerae.* ^c^ based on analogues in [89] clinical breakpoints for *Vibrio* spp. including *Vibrio cholerae.*

## Supplementary Materials

The following are available online at https://www.mdpi.com/2076-0817/9/12/1051/s1, Figure S1: in-vitro lysis of vB_VhaM_pir03 against 31 *Vibrio* spp. strains at multiplicity of infections (MOI) 0.1, 1 and 10 for 18 h. Bacterial growth was indicated by the absorbance (OD_600_) value. SE bars were shown for the mean of n = 3.
