# Supplementary figures and images for: Biological and Genomic Characterization of a Novel Jumbo Bacteriophage, vB_VhaM_pir03 with Broad Host Lytic Activity against Vibrio harveyi
Source: Pathogens. 2020 Dec 15;9(12):1051. doi: 10.3390/pathogens9121051 (PMC7765460; doi:10.3390/pathogens9121051)

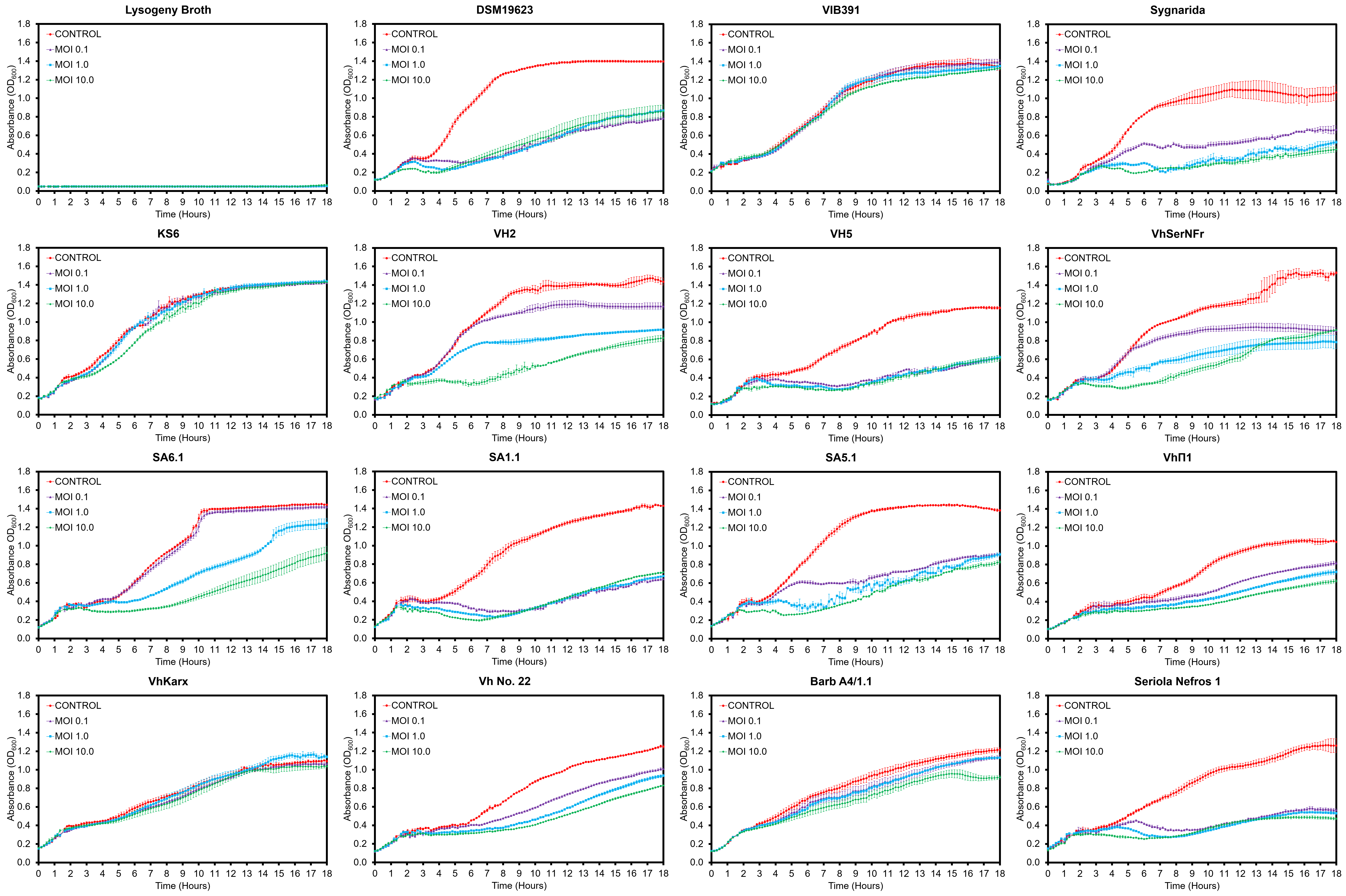

Supplement: Supplementary file 1 [file pathogens-09-01051-s001.zip › Supplementary/S1_1.png]

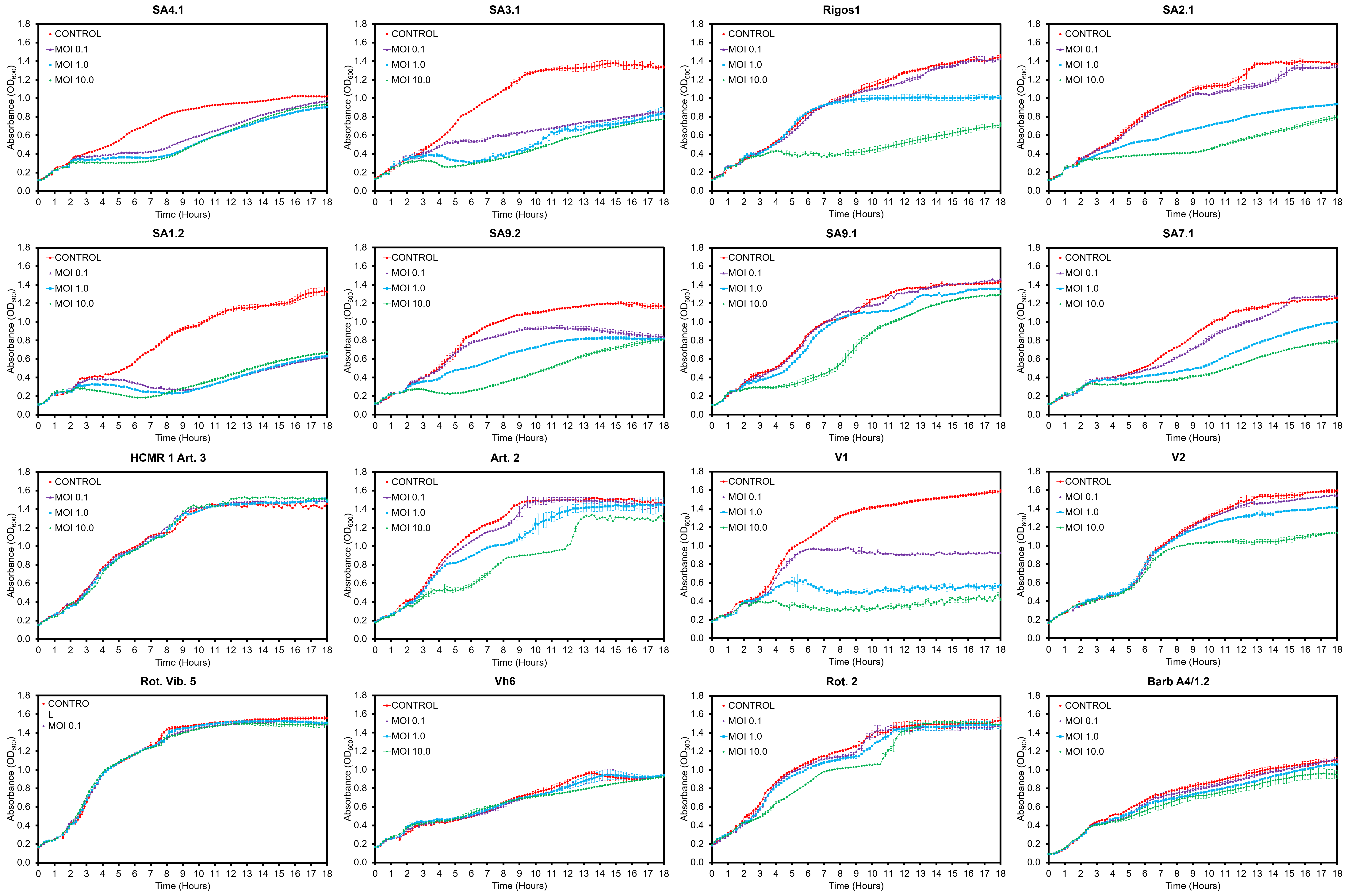

Supplement: Supplementary file 1 [file pathogens-09-01051-s001.zip › Supplementary/S1_2.png]
